# Pan-Ras-In-1 triggers cell wall lysis to eradicate *Staphylococcus aureus*

**DOI:** 10.1016/j.isci.2026.117291

**Published:** 2026-08-28

**Authors:** Weihua Han, Yinjuan Guo, Li Shen, Yu Huang, Xinru Yuan, Bingjie Wang, Can Yang, Rongrong Hu, Yongpeng Shang, Fangyou Yu

**Affiliations:** 1 Department of Clinical Laboratory, Shanghai Pulmonary Hospital, School of Medicine, Tongji University, Shanghai, China; 2 Shanghai Institute of Immunity and Infection Chinese Academy of Science, Shanghai, China

**Keywords:** *Staphylococcus aureus*, cell wall, Pan-Ras-In-1, programmed cell death, CidA, TarA

## Abstract

Given the global crisis of antimicrobial resistance, the dual anticancer and antibacterial properties of certain agents have attracted increasing attention as a potential alternative to conventional antimicrobial approaches. In this study, we characterized the bactericidal activity of an anticancer molecule (Pan-Ras-In-1) against *Staphylococcus aureus*, a pathogen associated with refractory infection. *In vitro* assays revealed that Pan-Ras-In-1 has a more pronounced bactericidal effect on *S. aureus* cells than the traditional antibiotics vancomycin and levofloxacin. Furthermore, Pan-Ras-In-1 displayed potential therapeutic efficacy *in vivo*, as assessed using the murine systemic infection and skin abscess models, suggesting its potential for subsequent clinical applications. Exposure of *S. aureus* to Pan-Ras-In-1 activates CidA-associated peptidoglycan hydrolase systems, which partially mediate bacterial programmed cell death. Peptide-centric local stability analyses revealed that Pan-Ras-In-1 binds to TarA protein, suppressing wall teichoic acid polymer production. The combined action of two mechanisms enables Pan-Ras-In-1 to compromise bacterial cell wall integrity.

## Highlights

• Pan-Ras-In-1 outperforms vancomycin/levofloxacin against extra- and intracellular *S. aureus*
• Pan-Ras-In-1 shows therapeutic efficacy in mouse systemic infection and skin abscess models
• Pan-Ras-In-1 activates CidA to trigger hydrolase and programmed cell death in *S. aureus*
• Pan-Ras-In-1 binds TarA specifically to inhibit wall teichoic acid polymer synthesis

## Teaser

Microbiology; Molecular biology

## Introduction

The widespread use of medical devices is often associated with the development of nosocomial infection among cancer patients, a population particularly vulnerable due to their immunocompromised states.^1^^,^^2^^,^^3^ Research has demonstrated that the organisms causing intravascular device-associated bacteremia are predominantly Gram-positive, with *Staphylococcus* species isolated most often.^1^ *Staphylococcus aureus*, a common Gram-positive human pathobiont, is a major cause of infection-related mortality and is responsible for severe invasive infections, including nosocomial pneumonia, endocarditis, osteomyelitis, and bloodstream infections.^4^ Due to antibiotic abuse, the emergence of methicillin-resistant *S. aureus* (MRSA) strains complicates clinical management of these fatal infections.^5^ Notably, treatment failures occur with particularly high frequency among cancer patients,^6^^,^^7^ urging a new therapeutic avenue against *S. aureus*.

In certain respects, bacterial pathogens share similarities with cancer cells; both can disseminate and induce tissue damage, and both exhibit rapid proliferation and metabolic rates.^8^ Furthermore, the eradication of bacterial pathogens or cancer cells requires immune activation. Therefore, it is plausible that specific therapeutic agents that suppress cancer progression may also show antimicrobial properties. In this study, we found that a pan-RAS inhibitor 3144 (Pan-Ras-In-1) exhibited efficient bactericidal activity against *S. aureus*. Pan-Ras-In-1 is a preclinical lead compound that directly targets RAS proteins (HRAS, KRAS, and NRAS), demonstrating potent antitumor efficacy.^9^ The tumor-eradicating activity of Pan-Ras-In-1 has been documented in animal cancer models, and no relevant adverse effects were observed at therapeutically active doses.^9^^,^^10^

*S. aureus* is surrounded by a thick cell wall that plays vital roles in maintaining cell integrity.^11^ Hence, the bacterial cell wall is a preferable target for developing new drugs for contemporary antimicrobial therapy.^12^ The peptidoglycan (PG) layer is the primary cell wall component that is composed of alternative *N*-acetylglucosamine (GlcNAc) and *N*-acetylmuramic acid (MurNAc) groups connected through β-1,4 glycosidic linkages, which are subsequently crosslinked by short peptide stems.^13^ The peptidoglycan network is a highly dynamic structure that is maintained not only by the synthesis and modification of new strands but also by the cleavage and degradation of existing strands.^14^ *S. aureus* contains no less than 18 different peptidoglycan hydrolases (PGHs) to cleave specific sites in the PG.^14^ Several direct and indirect mechanisms involved in the regulation of PGHs are known to exist. Importantly, the *cidABC operon* facilitates PGH regulation at the post-transcriptional level by controlling the transport of PGHs across the membrane.^15^ A putative holin encoded by *cidA* positively influences murein hydrolase activity, thereby disrupting membrane integrity and promoting bacterial cell death.^16^^,^^17^ On the other hand, wall teichoic acid (WTA) has been recognized as another essential component of the cell wall architecture in Gram-positive bacteria and plays critical roles in mediating drug resistance.^11^ WTA biosynthesis is a complex process that involves the sequential action of several enzymes, including *N*-acetylglucosamine-1-phosphate transferase (TarO) and *N*-acetylmannosamine transferase (TarA), which are involved in the initial steps of WTA polymer production.^18^ Disruption of WTA polymer assembly and export in *S. aureus* leads to lethal consequences.^18^^,^^19^ Therefore, WTA biosynthesis represents a theoretically promising target for new antibiotic development.

In this study, we found that the novel anti-cancer agent Pan-Ras-In-1 exhibited noticeable bactericidal activity against *S. aureus*. We verified that Pan-Ras-In-1 kills *S. aureus* by activating PGHs via a *cidA*-dependent pathway, thereby disrupting cell wall integrity. Furthermore, the *tarA*.13, serves as the binding target of Pan-Ras-In-1, then disrupts WTA biosynthesis. Notably, the results of mouse infection models further confirmed the bactericidal activity of Pan-Ras-In-1 *in vivo*. The findings of this study provide a novel therapeutic approach to address the antimicrobial resistance (AMR) dilemma in *S. aureus* infections.

## Results

### Pan-Ras-In-1 possessed bactericidal activity against *S. aureus in vitro*

**Figure 1:**
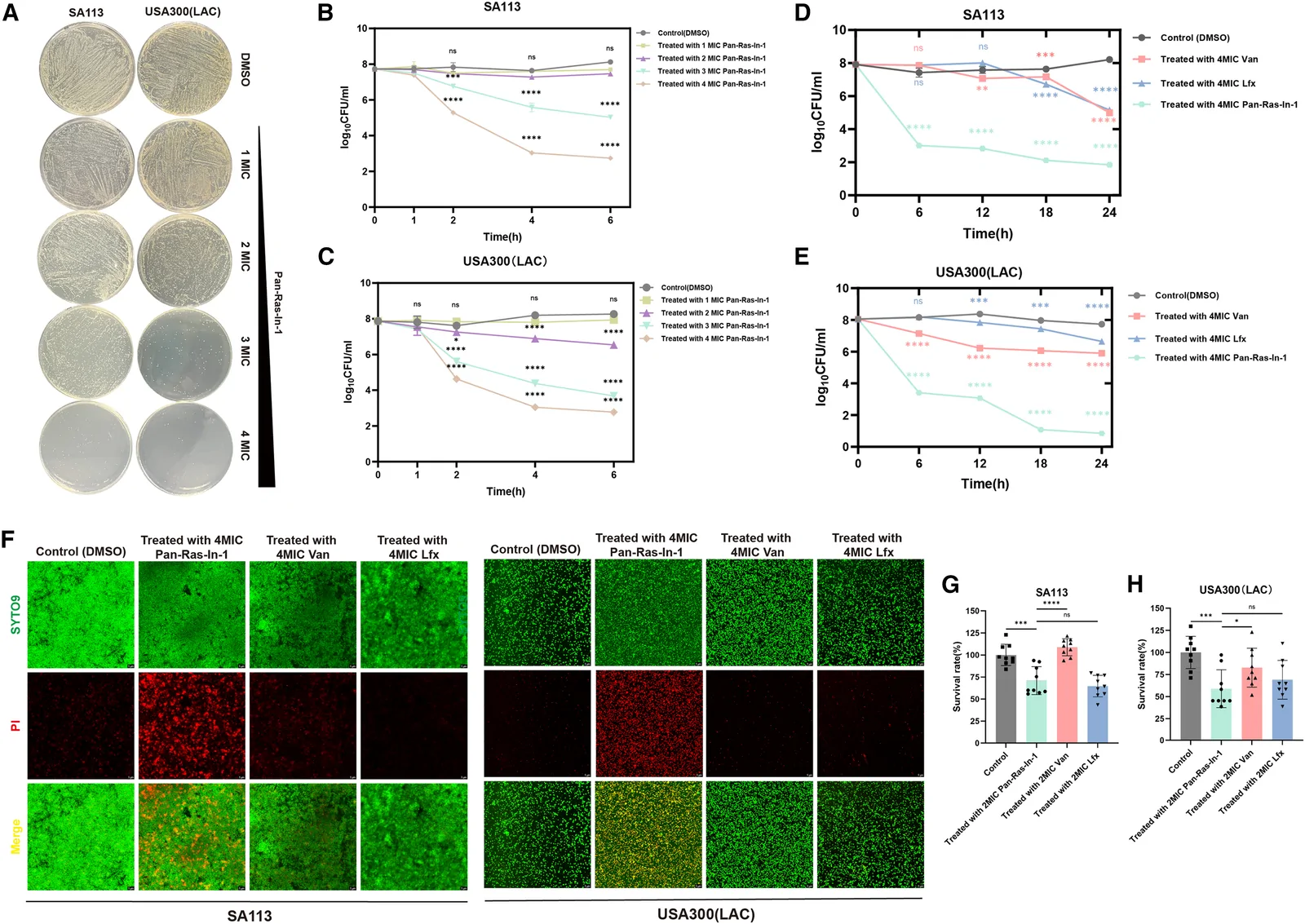
*In vitro* bactericidal property of Pan-Ras-In-1 (A) Representative images demonstrating the bactericidal effects of Pan-Ras-In-1 after 2-h exposure. (B and C) The concentration-dependent bactericidal efficiency of Pan-Ras-In-1 against *S. aureus* (*n* = 4). (D and E) The 24-h time-kill curves of Pan-Ras-In-1, vancomycin, and levofloxacin at concentrations of 4 × MIC (*n* = 4). (F) Fluorescent images of live (green)/dead (red) bacterial staining. Scale bars, 5 μm. (G and H) Activity of Pan-Ras-In-1, vancomycin, and levofloxacin against intracellular *S. aureus* in Raw 264.7 murine macrophages (*n* = 9). One-way analysis of variance (ANOVA) was used to analyze the statistical difference between multiple groups after passing the normal distribution test (B, C, D, E, G, and H). Error bars in the figures represent the standard deviation of the dataset (mean ± standard deviation). ns, not significant, ∗*p* < 0.05, ∗∗*p* < 0.01, ∗∗∗*p* < 0.001, ∗∗∗∗*p* < 0.0001.

Patients with cancer are significantly immunosuppressed and more likely to experience bacterial infections.^20^ In this study, our results revealed that Pan-Ras-In-1, a previously reported anticancer small molecule, unexpectedly exhibited prominent bactericidal activity against *S*. *aureus* during our compound screening for toxin release inhibitors, with minimum inhibitory concentrations (MICs) ranging from 2.25 to 4.5 μg/mL (3.125–6.25 μM) across MSSA and MRSA strains (Table S1). Besides, the minimum bactericidal concentrations (MBCs) of Pan-Ras-In-1 for *S. aureus* ranged from 2.25 to 9 μg/mL (3.125–12.5 μM) (Table S1). Given that the ratio of MBC to MIC was ≤4,^21^ Pan-Ras-In-1 is a potential bactericidal molecule against *S. aureus*. Next, time-kill kinetics assays were performed to evaluate the bactericidal activity of Pan-Ras-In-1. As shown in Figures 1A–1C, there was a dose-dependent killing efficiency to eradicate the MSSA strain SA113 and the MRSA strain USA300 (LAC). The viable SA113 cells reduced from 7.73 ± 0.09 to 2.74 ± 0.06 log_10_ colony-forming units (CFU)/mL under 6 h treatment of 4 × MIC Pan-Ras-In-1 (Figure 1B). Moreover, 4 × MIC Pan-Ras-In-1 eradicated viable USA300 (LAC) cells from 7.86 ± 0.16 to 2.77 ± 0.07 log_10_ CFU/mL after 6 h (Figure 1C), indicating Pan-Ras-In-1 exhibited a promising bactericidal effect for *S. aureus in vitro*. However, the MBC values of Pan-Ras-In-1 against *Klebsiella pneumonia*, *Pseudomonas aeruginosa*, and *Acinetobacter baumannii* were all above 18 μg/mL (25 μM) (Table S1; Figure S1), indicating Pan-Ras-In-1 was not a broadly applicable bactericidal molecule against Gram-negative organisms.

### Pan-Ras-In-1 achieved better bactericidal effect against extracellular or intracellular *S. aureus* than vancomycin and levofloxacin *in vitro*

The bactericidal performance of Pan-Ras-In1-1 was further evaluated by comparing it with levofloxacin and vancomycin *in vitro*. *S. aureus* SA113 exhibited sensitivity to vancomycin and levofloxacin with MICs of 1 μg/mL and 0.06 μg/mL, respectively (Figure S2). *S. aureus* USA300 (LAC) showed sensitivity to vancomycin (MIC, 0.5 μg/mL), but resistance to levofloxacin (MIC, 4 μg/mL) (Figure S2). As Figure 1D showed, 4 × MIC vancomycin and 4 × MIC levofloxacin caused 3.21 ± 0.03 and 3.05 ± 0.05 log_10_ CFU/mL reduction of *S. aureus* SA113 cells after 24 h, respectively. In contrast, the viable culture eradicated by Pan-Ras-In-1 was 6 ± 0.07 log_10_ CFU/mL at concentrations of 4×MIC (Figure 1D). For the eradication of the MRSA strain USA300 (LAC), Pan-Ras-In-1 also demonstrated greater bactericidal activity than vancomycin and levofloxacin. Figure 1E shows that a 6.88 ± 0.05 log10 CFU/mL reduction in viable cells was observed after 24 h of exposure to 4 × MIC Pan-Ras-In-1. In contrast, after exposure to viable *S. aureus* USA300 (LAC) cells with vancomycin and levofloxacin at 4 × MIC for 24 h, only 1.83 ± 0.15 and 1.08 ± 0.03 log10 CFU/mL reduction was observed, respectively (Figure 1E). What is more, we used a live/dead bacterial staining assay to further unveil the difference in bactericidal activity between vancomycin, levofloxacin, and Pan-Ras-In-1. A large proportion of *S. aureus* cells exhibited death following 4 h treatment with Pan-Ras-In-1 at 4×MIC, as indicated by positive red PI staining (Figure 1F). Meanwhile, a minimal amount of red-stained bacteria was observed in the vancomycin and levofloxacin-treated groups (Figure 1F), further confirming the potent bactericidal activity of Pan-Ras-In-1.

The intracellular habitat of *S. aureus* and the inadequate cellular penetration of antibiotics call for molecules with intracellular activity against *S. aureus*.^22^ Here, we assessed the intracellular bactericidal activity of Pan-Ras-In-1 in the murine macrophage cell line Raw264.7. Pan-Ras-In-1 exhibited intracellular bactericidal activity, as evidenced by a significant reduction in intercellular bacterial load at 2×MIC treatment (survival rates were 70.96% ± 14.87% for the SA113 strain [*p* < 0.001] and 58.99% ± 20.12% for the USA300 (LAC) strain [*p* < 0.001]) compared with the DMSO control group (Figures 1G and 1H). This effect was more pronounced than that of vancomycin [survival rates were108.69% ± 9.00% for the SA113 strain and 82.73% ± 20.65% for the USA300 (LAC) strain] and comparable with levofloxacin (survival rates were 64.75% ± 11.52% for the SA113 strain and 69.06% ± 20.70% for the USA300 (LAC) strain) (Figures 1G and 1H). Overall, these results indicated that Pan-Ras-In-1 is more potent than vancomycin and levofloxacin in clearing *S. aureus* cells.

### Pan-Ras-In-1 exerts an inhibitory effect on biofilm development

**Figure 2:**
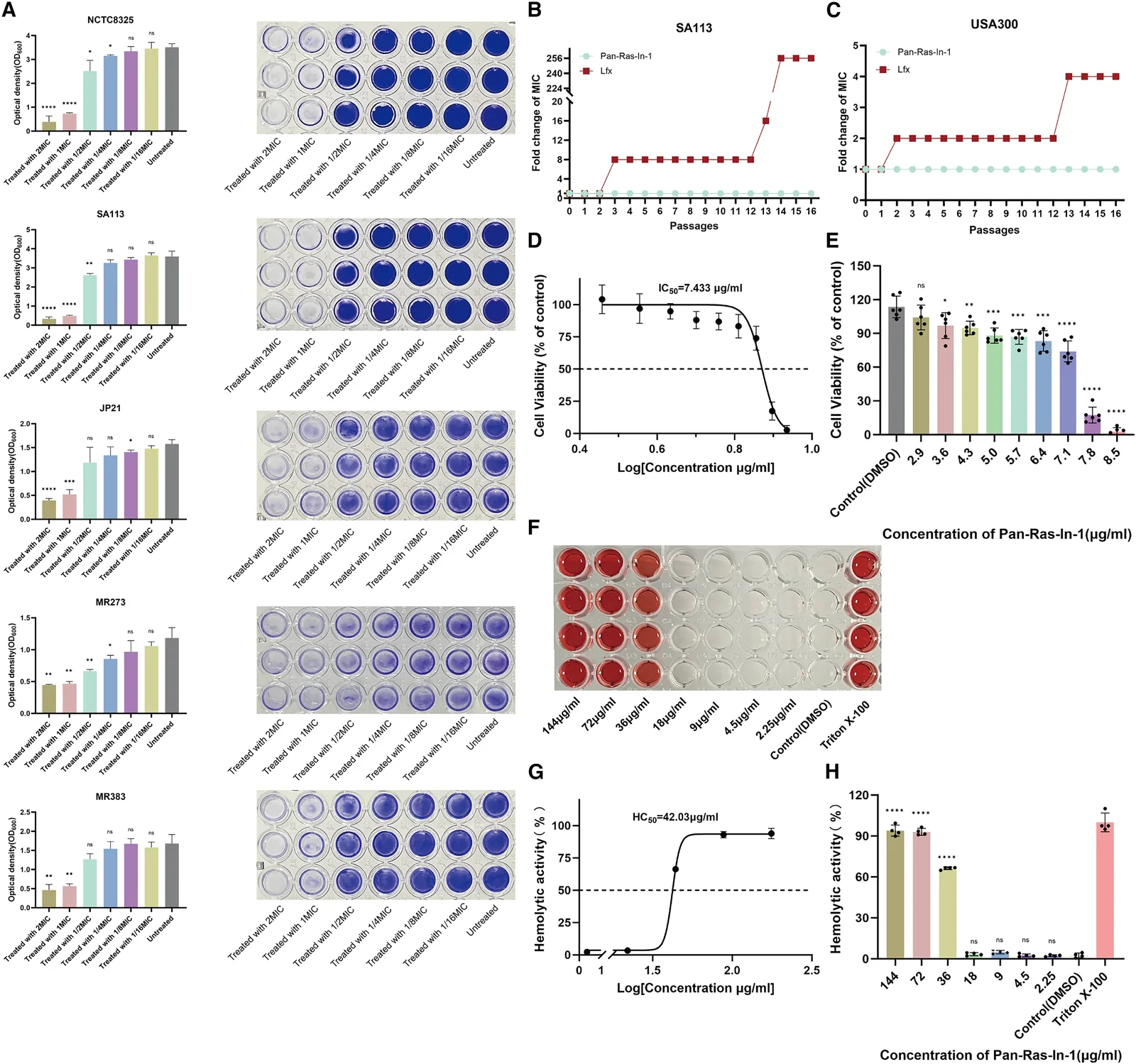
Assessments of the safety and tolerability of Pan-Ras-In-1 *in vitro* (A) The biofilm formation ability of *S. aureus* treated with Pan-Ras-In-1 on polypropylene 96-well plates (right), and the optical density values at 600 nm were measured after the biofilms were dissolved with acetic acid (left) (*n* = 3). (B and C) Induction of resistance in *S. aureus* after continuous exposure to Pan-Ras-In-1 and levofloxacin at 1/2 MIC levels. (D) The concentration of Pan-Ras-In-1 that resulted in a 50% reduction in Raw264.7 cell viability (IC50) was determined by generating a standard curve. (E) The viability of Raw264.7 cells after being treated with Pan-Ras-In-1 (*n* = 6). (F) Representative images of red cell hemolysis in response to Pan-Ras-In-1. (G) The concentration of Pan-Ras-In-1 resulting in 50% red cell lysis (HC_50_) was measured by generating the standard curve. (H) The viability of human red cells after being treated with Pan-Ras-In-1 (*n* = 4). A two-tailed Student’s *t* test was used to analyze the statistical difference between two groups after passing the normal distribution test (A, E, and H). Error bars in the figures represent the standard deviation of the dataset (mean ± standard deviation). ns, not significant, ∗*p* < 0.05, ∗∗*p* < 0.01, ∗∗∗*p* < 0.001, ∗∗∗∗*p* < 0.0001.

The ability of *S. aureus* to adhere to biotic and abiotic surfaces and to form biofilm, is at the root of many persistent and chronic bacterial infections.^23^ What is important to note that antibiotics used at sub-inhibitory concentration (sub-MIC) are always associated with increased biofilms formation ability of *S. aureus*.^24^ To assess the potential risk of enhanced biofilm formation at sub-MIC concentrations, we evaluated the biofilm-forming capacity of five strong biofilm-producing *S. aureus* strains when exposed to gradient concentrations of Pan-Ras-In-1. As shown in Figure 2A, *S. aureus* exhibited a biofilm-defective phenotype after treatment with above-inhibitory concentrations of Pan-Ras-In-1. What stand out in Figure 2A were the diminished biofilm-forming abilities of NCTC8325, SA113 and MR273 strains under the treatment of sub-MIC Pan-Ras-In-1 (NCTC8325: OD_600_ reduced from 3.51 ± 0.12 to 2.52 ± 0.36 and 3.15 ± 0.03 at 1/2 MIC and 1/4 MIC, respectively; SA113: OD_600_ reduced from 3.60 ± 0.23 to 2.62 ± 0.07 at 1/2 MIC; MR273: OD_600_ reduced from 1.18 ± 0.13 to 0.66 ± 0.02 and 0.86 ± 0.04 at 1/2 MIC and 1/4 MIC, respectively). Thus, these results suggest that Pan-Ras-In-1 can suppress biofilm formation.

### *S. aureus* does not develop drug resistance to Pan-Ras-In-1

Given the global escalation of AMR, it is imperative to evaluate the propensity of a newly developed bactericidal agent to develop resistance. In this study, we assess the propensity of *S. aureus* SA113 and USA300 (LAC) to develop resistance to Pan-Ras-In-1 following serial exposure at 1/2 MIC, with levofloxacin as a control. After 16 passages, the MIC of levofloxacin for SA113 and USA300 (LAC) increased 256-fold and 4-fold, respectively. In contrast, the MIC of the novel bactericidal agent Pan-Ras-In-1 was not altered during the same period (Figures 2B and 2C), suggesting that Pan-Ras-In-1 may represent a valuable option for tackling drug resistance.

### Pan-Ras-In-1 imparted no toxicity to eukaryotic cells at effective bactericidal concentrations

Given that cytotoxic effects would hinder the clinical application of bactericidal compounds, we assessed the cytotoxicity of Pan-Ras-In-1. Notably, the CCK-8 assay results revealed that Pan-Ras-In-1 did not affect the proliferation of the murine macrophage cell line Raw264.7 at effective bactericidal concentrations (IC50 = 7.433 μg/mL) with selectivity index (SI) 3.30 (Figures 2D and 2E). What’s more, Pan-Ras-In-1 at effective bactericidal concentrations exhibited low hemolytic activity toward healthy human red blood cells (Figure 2F), exhibiting a favorable HC_50_ index (HC_50_ = 43.02 μg/mL) and selectivity index (SI = 18.68) (Figures 2G and 2H). Therefore, Pan-Ras-In-1 appears to be a promising candidate with acceptable safety, though further structural optimization is required to reduce toxicity for further clinical applications.

### The transcriptome profile of *S. aureus* treated with Pan-Ras-In-1

**Figure 3:**
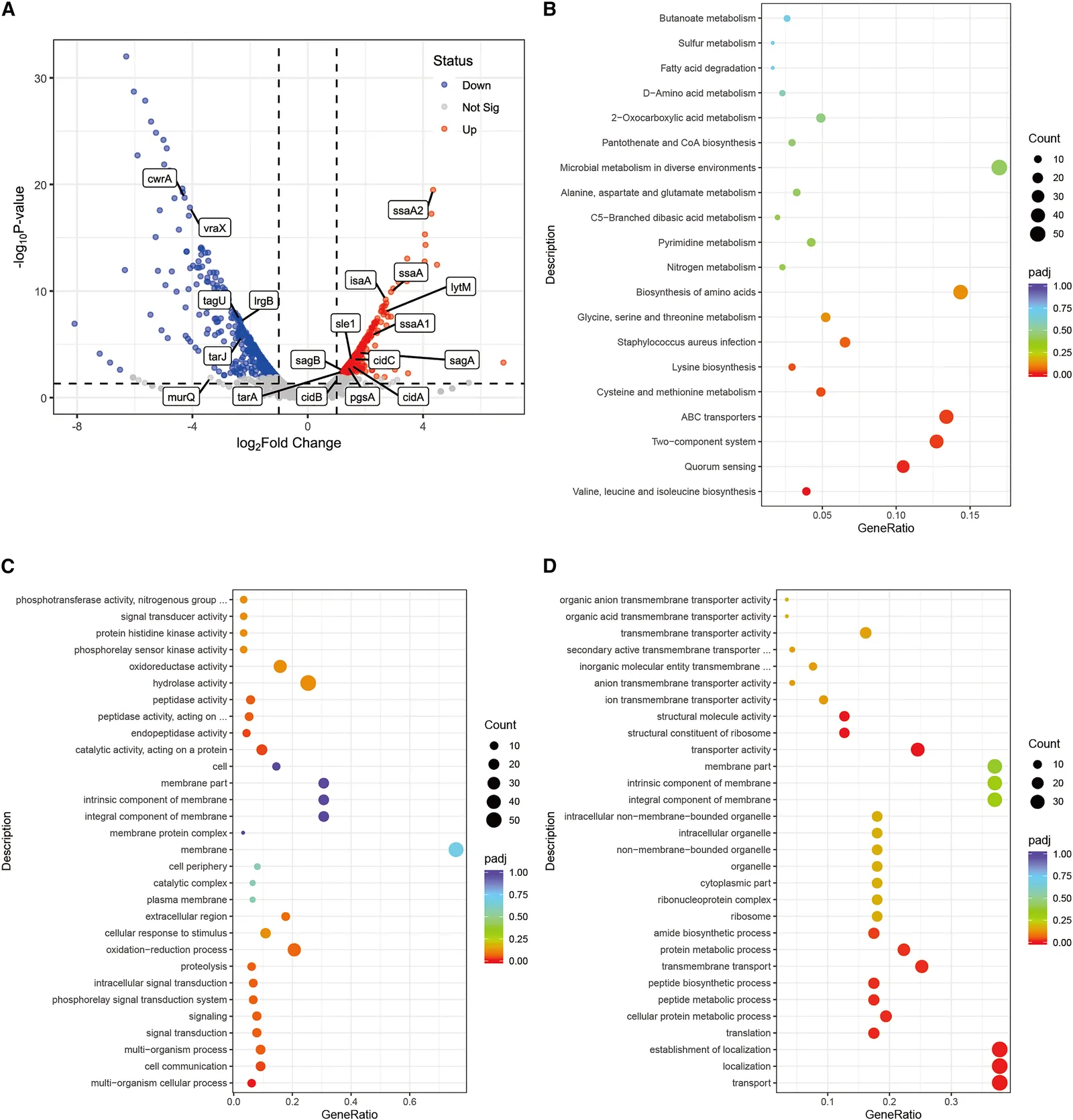
RNA-seq analysis of *S. aureus* SA113 treated with Pan-Ras-In-1 (A) Volcano plot analyses of total differential expression genes (DEGs) of *S. aureus* SA113 after treatment with Pan-Ras-In-1. Each dot represents an open reading frame, with upregulated genes shown in red and downregulated genes shown in blue. False discovery rate (FDR) ≤ 0.05 and |log_2_(fold change)| ≥ 1 were set as the threshold for significantly differential expression. (B) Kyoto Encyclopedia of Genes and Genomes (KEGG) enrichment pathway analysis of DEGs. (C) Gene Ontology (GO) enrichment pathway analysis of down-regulated DEGs. (D) GO enrichment pathway analysis of up-regulated DEGs.

To further explore the effect of Pan-Ras-In-1 on *S. aureus*, RNA-Seq was performed. A total of 818 differentially expressed genes (DEGs) were identified according to the threshold (false discovery rate [FDR] ≤ 0.05 and |log_2_(fold change)| ≥ 1), with 370 significantly up-regulated genes and 448 significantly down-regulated genes compared with the DMSO control (Figure 3A). The Kyoto Encyclopedia of Genes and Genomes (KEGG) enrichment analysis identified genes enriched considerably for microbial metabolism in diverse environments, ABC transporters, two-component systems, and Quorum sensing (Figure 3B). ABC transporters are associated with bacterial physiological functions, including the import of essential nutrients and the export of toxic proteins.^25^ The alterations in metabolic processes indicated that *S. aureus* cells repair physiological functions in response to damage caused by Pan-Ras-In-1. In addition, Gene Ontology (GO) enrichment analysis revealed that the downregulated genes were primarily associated with hydrolase activity and oxidoreductase activity (Figure 3C), whereas the upregulated genes were involved in transmembrane transport, membrane components, and metabolic process (Figure 3D). Consistent with enrichment results, multiple genes associated with cell membrane formation were identified. The genes encoding known or putative PGHs were significantly upregulated in the Pan-Ras-In-1-treated group, including *ssaA*, *sle1*, *lytM*, *isaA*, *sagA*, and *sagB* (Figure 3A; Table S2). Furthermore, the genes involved in PGHs regulatory systems were also influenced by Pan-Ras-In-1. For example, the *cidABC* operon, which functions as a positive regulator of autolysis, was significantly upregulated (Figure 3A; Table S2).^15^ In turn, the negative regulators *lytS* and its downstream *lrgAB* operon were significantly down-regulated in the Pan-Ras-In-1-treated cells (Figure 3A; Table S3).^26^ Thus, it is reasonable to predict that the PGH systems were activated in *S. aureus* cells upon treatment with Pan-Ras-In-1. Besides, the reduced expression of genes involved in peptidoglycan synthesis (*murQ*, *murI*, *murA2*, and *murT*) and techoic acid biosynthesis (*tagB*, *tagC*, *tagU*, *tarJ*, and *tarI*) further suggested that Pan-Ras-In-1 targets cell wall integrity to mediate *S. aureus* cell death (Figure 3A; Table S3).^27^ Based on RNA-seq results, we further explored the bactericidal mechanism of Pan-Ras-In-1 on bacterial cell wall hydrolysis in more detail.

### Pan-Ras-In-1 eliminated *S. aureus* by disrupting cell wall integrity

**Figure 4:**
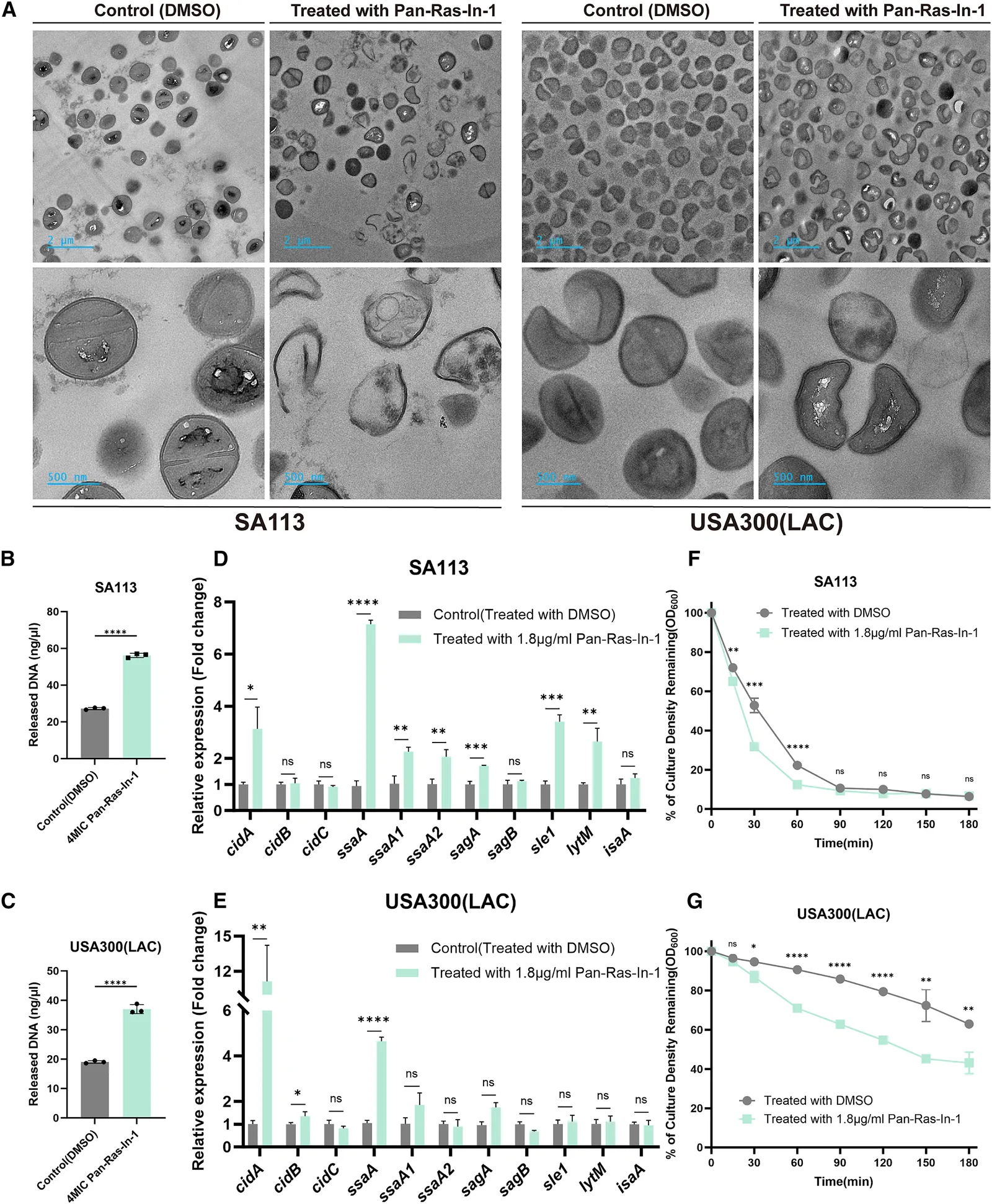
Pan-Ras-In-1 induces *S. aureus* cell death via cell wall disruption (A) TEM images of *S. aureus* SA113 and USA300 (LAC) incubated with DMSO or 4 × MIC Pan-Ras-In-1 for 4 h. Scale bars, 2 μm (above) and 500 nm (below). (B and C) The concentrations of leakage DNA in the supernatants of bacterial suspensions with treatments of DMSO or 4 × MIC Pan-Ras-In-1 for 4 h (*n* = 3). (D and E) Real-time fluorescence quantitative PCR (RT-qPCR) analysis for the transcription levels of the *cidABC* operon and PGH genes in *S. aureus* after treatment with 1.8 μg/mL Pan-Ras-In-1 (*n* = 3). (F and G) Autolysis activity of the *S. aureus* cells treated with 1.8 μg/mL Pan-Ras-In-1 or DMSO (*n* = 3). A two-tailed Student’s *t* test was used to analyze the statistical difference between two groups after passing the normal distribution test (B–G). Error bars in the figures represent the standard deviation of the dataset (mean ± standard deviation). ns, not significant, ∗*p* < 0.05, ∗∗*p* < 0.01, ∗∗∗*p* < 0.001, ∗∗∗∗*p* < 0.0001.

To verify the effect of Pan-Ras-In-1 on the *S. aureus* cell wall, TEM was used to observe the cell morphology. As shown in Figure 4A, Pan-Ras-In-1 treatment caused cell wall disruption in both SA113 and USA300 (LAC) strains. Meanwhile, the cells exhibited crumpled morphology with cytoplasmic leakage, indicating reduced cell wall rigidity (Figure 4A). Moreover, Pan-Ras-In-1-treated *S. aureus* cells exhibited significantly higher levels of extracellular DNA leakage than the DMSO control. After 4 h of treatment with 4 × MIC Pan-Ras-In-1, extracellular DNA levels increased from 27.23 ± 0.46 to 56.2 ± 0.94 ng/mL in *S. aureus* SA113 (Figure 4B). The MRSA strain USA300 (LAC), which possessed a thicker cell wall, showed an increase from 19.03 ± 0.37 to 37.03 ± 1.25 ng/mL compared with the control group (Figure 4C). Based on the clues RNA-seq provided, we hypothesized that active PGH systems might participate in cell wall disruption. To verify this, we primarily used Real-time fluorescence quantitative PCR (RT-qPCR) to further confirm the upregulation of PGH genes in Pan-Ras-In-1-treated bacterial cells. As shown in Figure 4D, genes encoding functional PGHs (including SsaA, SsaA1, SsaA2, SagA, Sle1, and LytM) were significantly upregulated in Pan-Ras-In-1-treated SA113 cells. The gene encoding the key PGH SsaA was also considerably upregulated in Pan-Ras-In-1-treated USA300 (LAC) cells (Figure 4E). Notably, we observed marked upregulation of the holin-encoding gene *cidA* in both SA113 and USA300 (LAC) strains (Figures 4D and 4E). Subsequently, we evaluated the autolysis capability by the Triton X-100-induced autolysis assay. Consistent with the upregulation of PGH-encoding genes, the stains treated with Pan-Ras-In-1 exhibited significantly enhanced autolysis activity compared with DMSO-treated controls (Figures 4F and 4G). Taken together, cell wall disruption serves as the primary mechanism for Pan-Ras-In-1-induced *S. aureus* death.

### Pan-Ras-In-1 activated PGHs activity in the CidA-dependent manner

**Figure 5:**
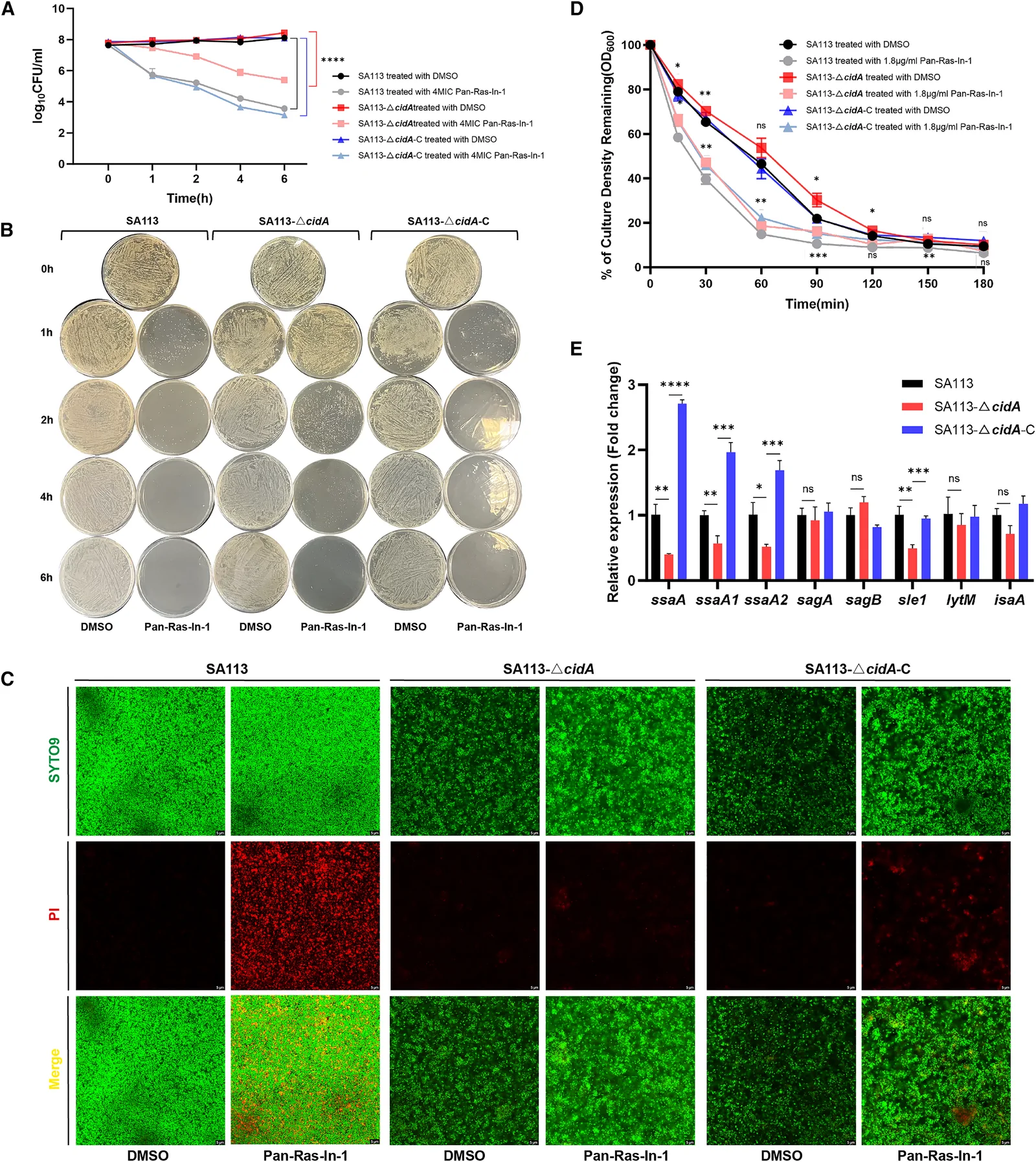
Pan-Ras-In-1 induces *S. aureus* cell death dependent on CidA (A) Bacterial killing kinetics of 4 × MIC Pan-Ras-In-1 against *S. aureus* SA113, SA113-△*cidA*, and SA113-△*cidA*-C (*n* = 4). (B) Representative images comparing the bactericidal effects of Pan-Ras-In-1 against *S. aureus* SA113, SA113-△*cidA*, and SA113-△*cidA*-C. (C) Fluorescent images of live (green)/dead (red) bacterial staining after treatment with Pan-Ras-In-1. Scale bars, 5 μm. (D) Autolysis of *S. aureus* SA113, SA113-△*cidA*, and SA113-△*cidA*-C after treatment with DMSO or 1.8 μg/mL Pan-Ras-In-1 (*n* = 3). (E) Real-time fluorescence quantitative PCR (RT-qPCR) analysis for the transcription levels of PGH genes in *S. aureus* SA113, SA113-△*cidA*, and SA113-△*cidA*-C (*n* = 3). A two-tailed Student’s *t* test was used to analyze the statistical difference between two groups (A and D), and one-way analysis of variance (ANOVA) was used to analyze the statistical difference between multiple groups (E) after passing the normal distribution test. Error bars in the figures represent the standard deviation of the dataset (mean ± standard deviation). ns, not significant, ∗*p* < 0.05, ∗∗*p* < 0.01, ∗∗∗*p* < 0.001, ∗∗∗∗*p* < 0.0001.

Our results suggest that CidA may be involved in *S. aureus* PCD induced by Pan-Ras-In-1. To verify this hypothesis, we constructed the *cidA* knockout and complemented mutants in the SA113 genetic background and compared their capacity to resist the bactericidal activity of Pan-Ras-In-1. Time-kill kinetic analysis revealed that 4×MIC Pan-Ras-In-1 treatment for 6 h resulted in reductions of 4.56 ± 0.04 and 4.94 ± 0.10 log_10_ CFU/mL in viable *S. aureus* SA113 and SA113-▵*cidA*-C cells, respectively (Figures 5A and 5B). Meanwhile, a 3.04 ± 0.06 log_10_ CFU/mL reduction was observed in SA113-▵*cidA* cells under the same treatment conditions (Figures 5A and 5B). The live/dead bacterial staining assay was also performed to visualize the bactericidal activity of Pan-Ras-In-1 against SA113, SA113-▵*cidA*, and SA113-▵*cidA*-C. Compared with the wild-type strain, SA113-▵*cidA* mutant exhibited enhanced resistance to Pan-Ras-In-1, as evidenced by far fewer PI-positive cells in the Pan-Ras-In-1-treated SA113-▵*cidA* group (Figure 5C). The *S. aureus* CidA protein is a membrane-associated molecule that controls cell death by promoting cell lysis and extracellular DNA release.^28^ Thus, we assessed the autolysis activity of these strains following treatment with sub-MIC Pan-Ras-In-1 or DMSO control. Compared with parental strain, the *cidA* knockout mutant exhibited decreased autolysis activity, and the reduced autolysis activity was restored in the *cidA* complemented strain (Figure 5D). Although the *cidA* knockout mutant also showed increased autolysis activity under Pan-Ras-In-1 treatment, the autolysis activity reduction in the wild-type strain was more pronounced than in the *cidA* knockout strain (Figure 5D). Besides, the *cidA* knockout mutant exhibited significantly reduced expression levels of *ssaA*, *ssaA1*, *ssaA2*, and *sle1* compared with the wild-type strain, indicating CidA functions as a positive regulator of SsaA family and Sle1 PGHs to induce autolysis (Figure 5E). These results suggested that functional CidA partially mediates the enhancement of Pan-Ras-In-1-induced autolysis activity.

Ten tolerant strains (SA113-1 to SA113-5 and USA300 (LAC)-1 to USA300 (LAC)-5) were generated after 16 serial passages of *in vitro* Pan-Ras-In-1 pressure induction; these isolates displayed enhanced tolerance to the bactericidal activity of Pan-Ras-In-1 without shifts in baseline MIC values (Figures S3A and S3B). We initially evaluated their ability to resist the bactericidal activity of Pan-Ras-In-1 relative to their control strains (SA113-TSB and USA300 (LAC)-TSB: living in the TSB without Pan-Ras-In-1 for 16 generations). When exposed to 4×MIC Pan-Ras-In-1 for 2 h, all induced strains derived from SA113 demonstrated significantly greater survival than the control strain SA113-TSB (cell viability: 0.77% ± 0.05% for SA113-TSB, 62.73% ± 6.98% for SA113-1, 28.26% ± 2.34% for SA113-2, 24.43% ± 2.03% for SA113-3, 8.36% ± 0.66% for SA113-4, and 2.86% ± 0.44% for SA113-5) (Figure S3C). Similarly, strains USA300 (LAC)-1, USA300 (LAC)-2, and USA300 (LAC)-5 exhibited notably enhanced resistance to Pan-Ras-In-1 killing compared with the USA300 (LAC)-TSB control strain (with cell viabilities of 21.48% ± 3.11%, 7.17% ± 1.37%, and 9.76% ± 0.47%, respectively, versus 3.75% ± 0.50% for the control) (Figure S3D). Since CidA was implicated as a potential Pan-Ras-In-1 target and drug-induced mutant genes frequently encode target proteins, we sequenced the *cidA* gene in all strains to identify potential mutation sites. A total of four single-nucleotide variants were detected (213_214insT, 241_242insT, 245_246insG, and 331G > C), resulting in a frameshift and a premature stop codon (Figures S3E and S3F). Notably, the 241_242insT mutation was present in all Pan-Ras-In-1-tolerant strains (USA300 (LAC)-1, -2, -5, and SA113-1, -2, -3, -4) causing the following codon misreading (Figures S3G–S3H). In contrast, this mutation was absent in non-tolerant strains USA300 (LAC)-3 and USA300 (LAC)-4, suggesting that 241_242insT may represent the key site to mediate tolerance to Pan-Ras-In-1.

### Pan-Ras-In-1 suppressed WTA synthesis in *S. aureus* by targeting the TarA protein

**Figure 6:**
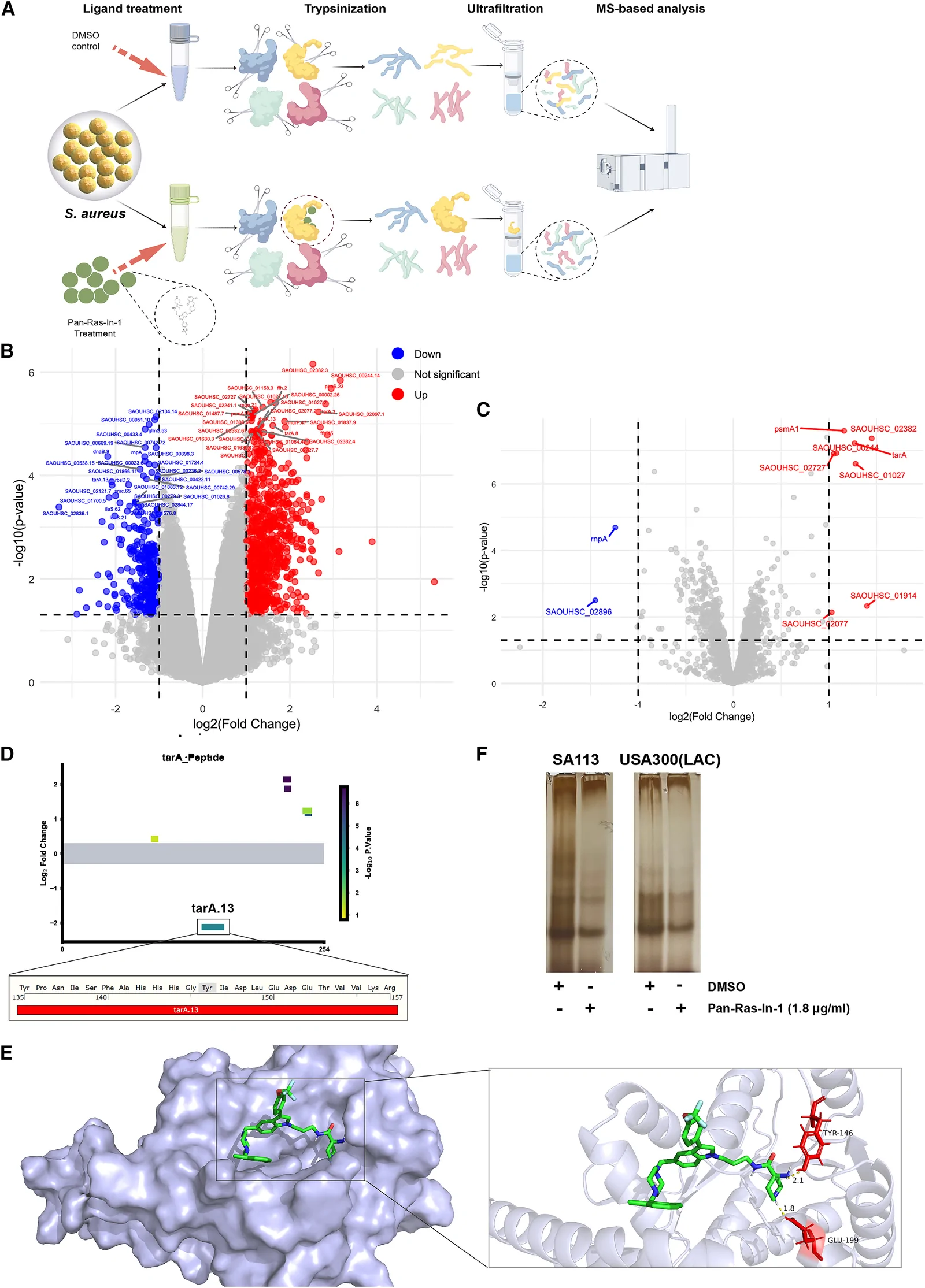
Pan-Ras-In-1 targets tarA.13 peptide to disrupt WTA biosynthesis (A) Workflow of PELSA. This figure was drawn by Figdraw (ID: WSOWAf4440). (B) Volcano plot visualization of all peptides from the PELSA analysis of SA113 lysates incubated with Pan-Ras-In-1. (C) Volcano plot visualization of protein levels. (D) Local stability profiles to reveal Pan-Ras-In-1-binding regions of TarA protein. The upper and lower boundaries of the gray shade correspond to log2foldChange values of 0.3 and −0.3, respectively. The *x* axis represents the protein sequence from the N-terminal to the C-terminal. (E) The molecular docking analysis between Pan-Ras-In-1 and TarA protein. The structure of the TarA protein was shown in slight purple, while Pan-Ras-In-1 was shown in green. The predicted binding residues were highlighted in red, and the hydrogen bonds were displayed in yellow. (F) Native PFGE profiles for WTA analysis.

The earlier-mentioned results demonstrate that the *cidA* knockout strain did not develop complete resistance to Pan-Ras-In-1’s bactericidal effect and still showed enhanced autolysis activity upon Pan-Ras-In-1 treatment. It seemed that Pan-Ras-In-1 is a multi-target bactericidal drug. To further validate the molecular targets of Pan-Ras-In-1, we employed the peptide-centric local stability assay (PELSA). This approach enables target protein identification while simultaneously mapping precise binding region.^29^ Based on the principle that ligand binding alters protein thermodynamic stability and proteolytic susceptibility, we digested proteins with excess trypsin, isolated the resulting peptides, and identified them through shotgun proteomics coupled with mass spectrometry (Figure 6A). In our quantitative proteomic analysis, we identified 772 significantly altered peptides in the Pan-Ras-In-1-treated group compared with the DMSO control group, comprising 172 peptides with decreased abundance and 600 peptides with increased abundance (Figure 6B). Additionally, 10 proteins exhibiting significant changes in abundance were identified, including 2 with decreased abundance and 8 with increased abundance (Figure 6C). Based on the PELSA results, TarA, a protein serving as *N*-acetylmannosamine transferase involved in *S. aureus* WTA biosynthesis,^18^ emerged as the promising functional target of Pan-Ras-In-1 (Figures 6B and 6C). As shown in Figure 6D, the abundance of peptide tarA.13 (residues 135–157) significantly reduced in the Pan-Ras-In-1 treated group (log2 FoldChange = −2.09, *p* < 0.01), suggesting that this specific region serves as the potential binding site for Pan-Ras-In-1. Subsequently, molecular docking analysis of Pan-Ras-In-1 and TarA was performed using AutoDock. Notably, the simulation identified two significant hydrogen-bond interactions: (1) a 2.1 Å hydrogen bond to TYR-146, which falls within tarA.13 peptide region, and (2) an additional 1.8 Å hydrogen bond with GLU-199 residue (Figure 6E). To further validate Pan-Ras-In-1’s inhibitory effect on WTA biosynthesis, we detected WTA polymer degrees in the *S. aureus* following Pan-Ras-In-1 treatment. As shown in Figure 6F, Pan-Ras-In-1 administration resulted in a significant reduction of WTA polymer content compared with the DMSO control groups. Taken together, our findings establish that Pan-Ras-In-1 disrupts WTA polymer production in *S. aureus* by specifically targeting the TarA protein.

### Pan-Ras-In-1 demonstrated significant efficacy against *S. aureus in vivo*

**Figure 7:**
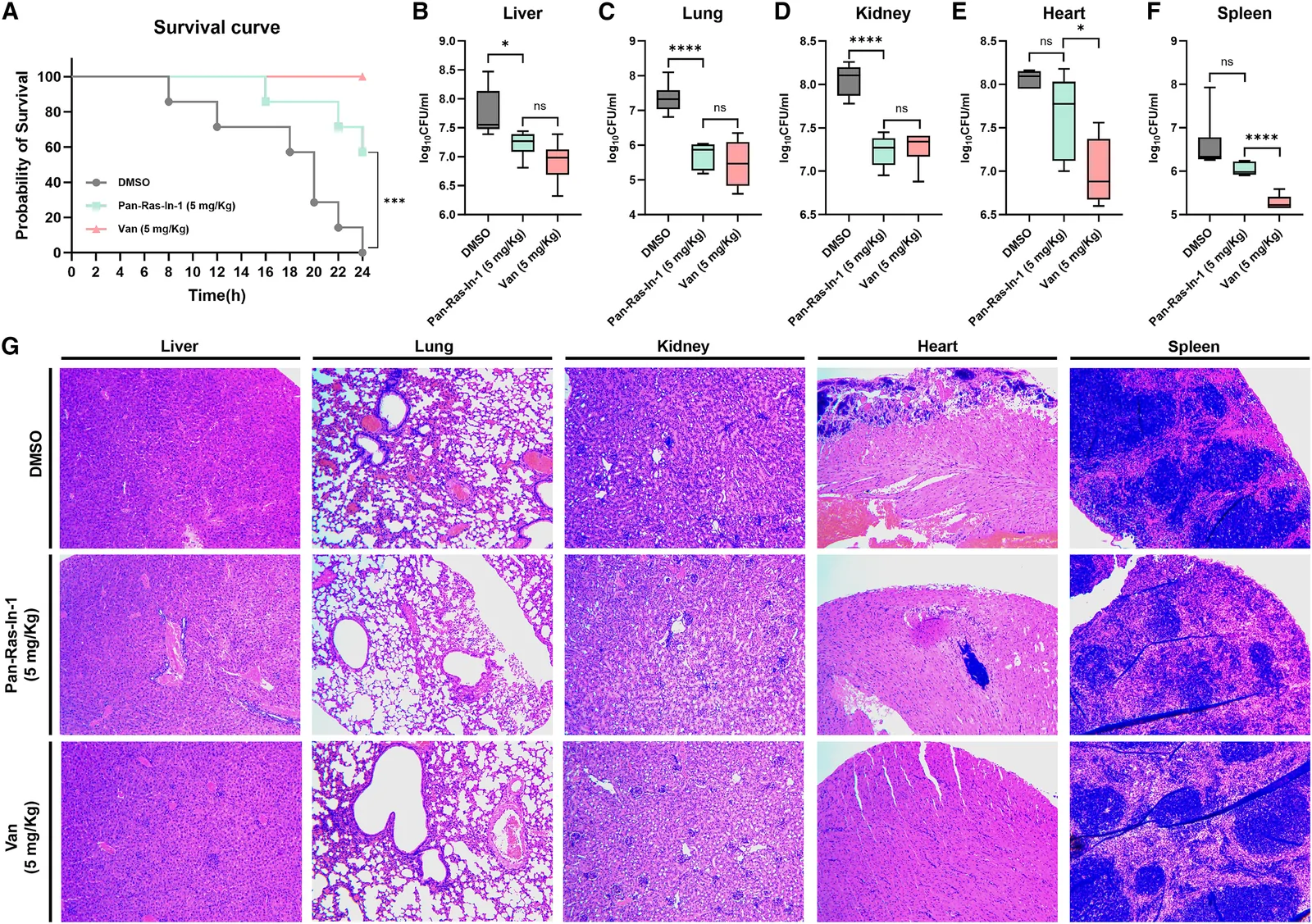
In vivo efficacy of Pan-Ras-In-1 in the murine systemic infection model (A) Survival rates of mice treated with DMSO, Pan-Ras-In-1, and vancomycin after being infected with *S. aureus* USA300 (LAC) (*n* = 7). (B–F) Bacterial load in liver, lung, kidney, heart, and spleen homogenates of respective groups (*n* = 6). (G) H&E staining of infected mice organs in respective treatment groups. Survival curves were generated using the Kaplan-Meier method, and intergroup comparisons were performed with the log-rank (Mantel-Cox) test (A). One-way analysis of variance (ANOVA) was used to analyze the statistical difference between multiple groups after passing the normal distribution test (B–F). Error bars in the figures represent the standard deviation of the dataset (mean ± standard deviation). ns, not significant, ∗*p* < 0.05, ∗∗*p* < 0.01, ∗∗∗*p* < 0.001, ∗∗∗∗*p* < 0.0001.

Encouraged by the promising *in vitro* antimicrobial activity of Pan-Ras-In-1, we used mice infection models to further characterize *in vivo* therapeutic efficacy. The mice bacteremia model has been largely used in research to evaluate new therapeutic treatments.^30^ In this study, we developed the mice bacteremia model, and equal Pan-Ras-In-1 or vancomycin was administered for treatment. As shown in Figure 7A, the 24-h survival rate of mice treated with Pan-Ras-In-1 was 57.14%, which was higher than that in the DMSO control group (0%) but lower than that of the group treated with vancomycin (100%). A significant difference (*p* < 0.001) was observed between the DMSO control group and the Pan-Ras-In-1-treated group. All mice in the DMSO control group died within 24 h post-infection; accordingly, surviving animals were euthanized at the 24-h time point for organ bacterial load quantification. After Pan-Ras-In-1 therapy, a substantial reduction in bacterial viability was observed in liver (reduce from 7.75 ± 0.38 to 7.22 ± 0.20 log_10_ CFU/mL, *p* < 0.01) (Figure 7B), lung (reduce from 7.35 ± 0.39 to 5.71 ± 0.35 log_10_ CFU/mL, *p* < 0.01) (Figure 7C), and kidney (reduce from 8.05 ± 0.16 to 7.24 ± 0.16 log_10_ CFU/mL, *p* < 0.01) (Figure 7D) compared with the DMSO control group. What’s more, the bacterial burden in these three organs was comparable between the groups treated with Pan-Ras-In-1 and vancomycin (Figures 7B–7D). However, no significant difference was observed in the bacterial burden of heart and spleen between the DMSO control group and the Pan-Ras-In-1 treated group (Figures 7E and 7F). Compared with organs harvested from DMSO-treated USA300 (LAC)-infected mice, tissues from Pan-Ras-In-1-treated animals exhibited markedly reduced infiltration of dominant inflammatory cells and diminished granulation tissue formation, particularly in the lung, kidney, and heart (Figure 7G). This finding demonstrates that Pan-Ras-In-1 exerts an inhibitory effect on *S. aureus*-mediated acute systemic inflammation response.

**Figure 8:**
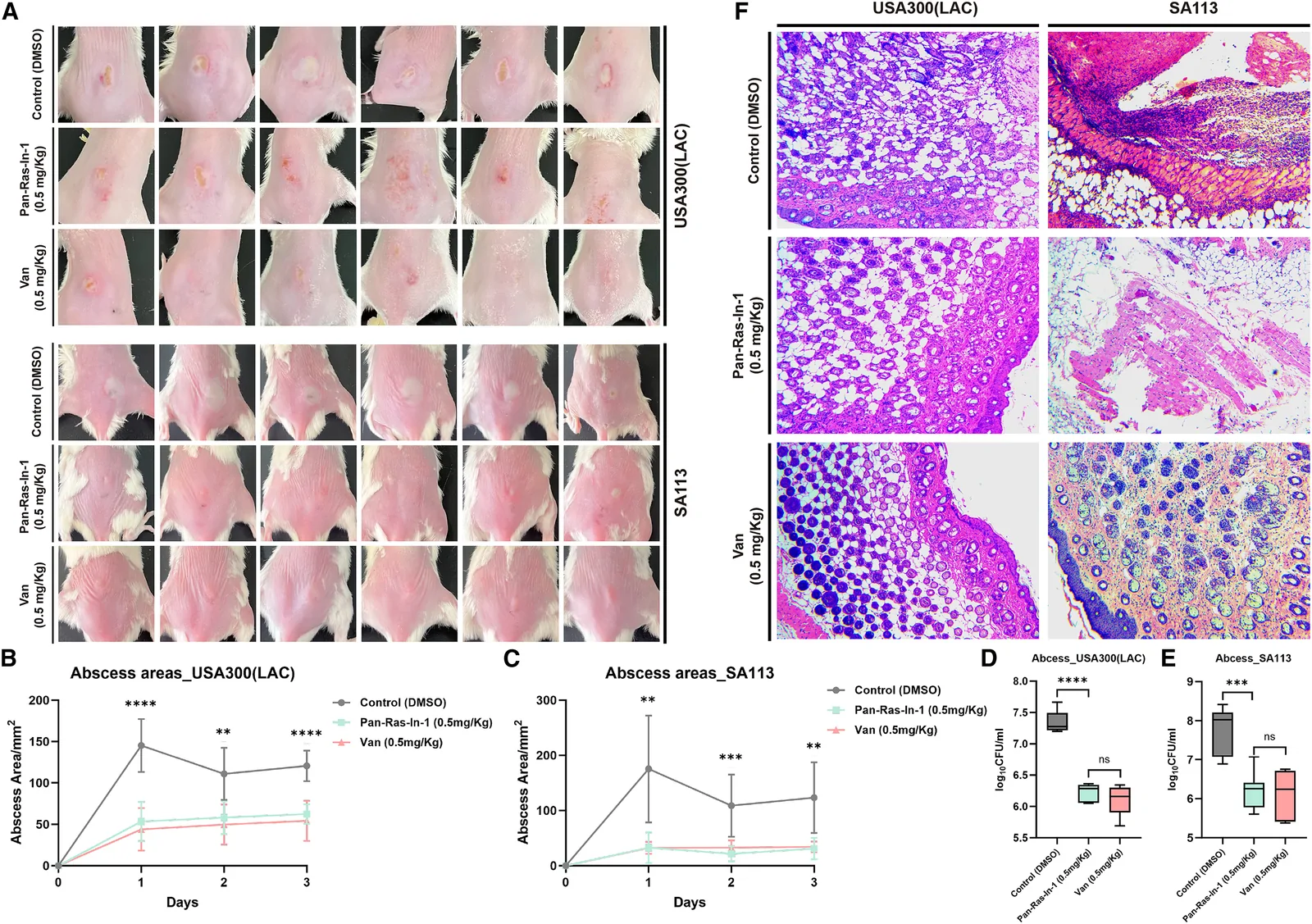
*In vivo* efficacy of Pan-Ras-In-1 in the murine skin abscess model (A) Photographs of the infection sites, with or without treatment, at first day. (B) The abscess areas were measured 3 days post-infected with USA300 (LAC) in all groups (*n* = 7). (C) The abscess areas were measured 3 days post-infected with SA113 in all groups (*n* = 8). (D) Bacterial load in abscess homogenates of mice infected with USA300 (LAC) (*n* = 6). (E) Bacterial load in abscess homogenates of mice infected with SA113 (*n* = 7). (F) The degree of lesion of the skin abscess was visualized by H&E staining. One-way analysis of variance (ANOVA) was used to analyze the statistical difference between multiple groups after passing the normal distribution test (B–E). Error bars in the figures represent the standard deviation of the dataset (mean ± standard deviation). ns, not significant, ∗*p* < 0.05, ∗∗*p* < 0.01, ∗∗∗*p* < 0.001, ∗∗∗∗*p* < 0.0001.

A murine skin abscess model was also performed to evaluate the efficiency of Pan-Ras-In-1 to prevent skin infection. Figure 8 Ashows that the control group without treatment developed pronounced skin abscesses. In contrast, the group treated with Pan-Ras-In-1 showed a significant reduction in abscess area, comparable to that observed in the vancomycin-treated group (Figure 8A). The quantitative findings revealed a pronounced prevention effect following administration of Pan-Ras-In-1. On day 3, the unhealed wound areas were 120.53 ± 17.22 and 123.31 ± 60.00 mm^2^ in mice infected with USA300 (LAC) and SA113, respectively, without treatment. In contrast, the mice treated with Pan-Ras-In-1 exhibited 62.36 ± 10.93 and 32.05 ± 18.36 mm^2^ skin abscess on day 3 after infected USA300 (LAC) and SA113, respectively (Figures 8B and 8C). The bacterial burden in the Pan-Ras-In-1-treated group was much lower than that in the control group but comparable with that in the vancomycin-treated group (USA300 (LAC): 7.35 ± 0.16 log_10_ CFU/mL for DMSO control group, 6.23 ± 0.13 log_10_ CFU/mL for Pan-Ras-In-1-treated group, 6.10 ± 0.22 log_10_ CFU/mL for vancomycin-treated group; SA113: 7.70 ± 0.59 log_10_ CFU/mL for DMSO control group, 6.22 ± 0.44 log_10_ CFU/mL for Pan-Ras-In-1-treated group, 6.14 ± 0.55 log_10_ CFU/mL for vancomycin-treated group) (Figures 8D and 8E). Interestingly, H&E histological staining showed that Pan-Ras-In-1 treatment greatly alleviated inflammatory cell infiltration in subcutaneous tissues, and this protective effect was more evident than that observed in the vancomycin-treated group (Figure 8F). These results further underscore the efficacy of Pan-Ras-In-1 in treating *S. aureus*-infected skin abscesses.

## Discussion

The global dissemination of antimicrobial-resistant *S. aureus* has emerged as an urgent public health concern, especially MRSA strains, which often cause severe invasive infections in immunocompromised patients. Oncology patients, a population that is vulnerable to nosocomial infections during prolonged hospitalization, are prone to antibiotic-associated adverse reactions.^6^ Therefore, special caution is warranted for novel therapeutic strategies to cure vital infections in cancer patients. In this study, we unexpectedly characterized an anticancer molecule that exhibited potent bactericidal activity against *S. aureus*. The MIC values of Pan-Ras-In-1 against both MSSA and MRSA strains ranged from 2.25 to 4.5 μg/mL, which were lower than the safety concentration determined by cytotoxicity assays. *In vitro*, Pan-Ras-In-1 exhibited more potent bactericidal activity against *S. aureus* compared with conventional antibiotics vancomycin and levofloxacin. Furthermore, mice infection models revealed Pan-Ras-In-1 therapeutic efficacy *in vivo*, suggesting that it is a promising candidate for further antibacterial drug development.

Initially, CidA gained prominence as a bacteriophage protein that promotes PCD, similar to the membrane-associated eukaryotic apoptotic Bcl-2 proteins.^31^ As a canonical holin, CidA forms a hole in the membrane and then allows cytoplasmic murein hydrolase to degrade peptidoglycan.^32^ In this study, we demonstrated that Pan-Ras-In-1 activated the *S. aureus* PGHs system in a CidA-dependent manner, ultimately leading to cell wall disruption through PGHs overstimulation. Previous studies have reported that CidA functions as a PCD regulator during biofilm formation in response to carbohydrate metabolism.^33^^,^^34^ The RNA-seq results presented in this study showed that Pan-Ras-In-1 markedly perturbed the metabolic state of *S. aureus* cells. Thus, it is intriguing to consider whether the altered metabolism served as a signal to stimulate CidA-dependent PGHs activation and PCD acceleration. However, the bactericidal effect of Pan-Ras-In-1 was not completely abolished in the *cidA*-knockout strain, demonstrating other potential proteins to be the targets of Pan-Ras-In-1.

WTA-deficient strains exhibited morphological abnormalities and increased autolysis.^35^^,^^36^^,^^37^ Schlag et al. reported that the WTA-lacking mutant (SA113 Δ*tagO*) was more susceptible to autolysis than the wild-type strain due to the higher binding capacity of autolysins to the cell wall.^37^ Moreover, they found that the loss of WTA affected PG cross-linking, indicating complex crosstalk between the WTA structure and PG components. In our study, we illustrated that the cell wall disruption induced by Pan-Ras-In-1 results from the synergistic action of two mechanisms: activation of PGH activity and inhibition of WTA biosynthesis. The essential enzyme TarA, involved in initial WTA polymer assembly, was shown to be a potential target of Pan-Ras-In-1 by PELSA assays, which also enabled detection of the specific peptide for Pan-Ras-In-1. WTA production and polymer assembly were significantly reduced in *S. aureus* cells treated with Pan-Ras-In-1. Therefore, it is plausible that the demolition of the WTA structure accelerated autolysis induced by activated PGHs, which may also explain why the *cidA* mutant demonstrated only partial resistance to the eradication effect and to the increased autolysis induced by Pan-Ras-In-1.

**Figure 9:**
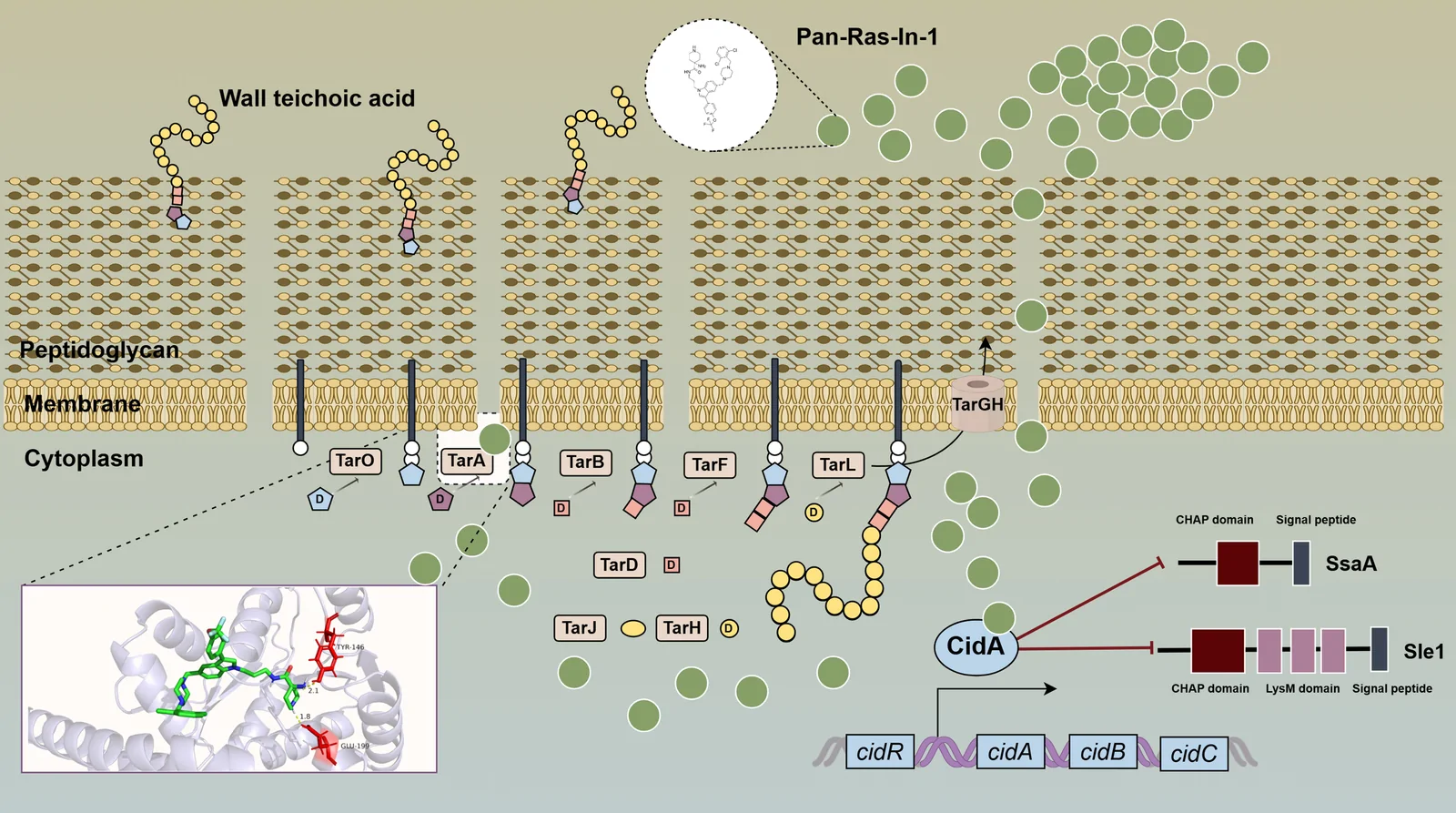
Schematic representation of the proposed mechanism of action for Ras-In-1 This figure was created using FigDraw (ID: ITSRSbb66e).

In conclusion, our results indicate that the anticancer agent Pan-Ras-In-1 exhibited noticeable bactericidal activity against *S. aureus*. Furthermore, we demonstrate that Pan-Ras-In-1-induced PCD in *S. aureus* occurs through the synergistic action of Cid-dependent PGHs activation and TarA-mediated inhibition of WTA polymer production, ultimately leading to cell wall disruption (Figure 9). This study is the first to demonstrate the role of Pan-Ras-In-1 in eliminating *S. aureus* infection and provides insight into the specific mechanism of Pan-Ras-In-1-triggered bacterial cell death. Given the results we reported, we believed that the application of Pan-Ras-In-1 in further clinical antibacterial therapy warranted further investigation.

### Limitations of the study

In this study, we performed assays to evaluate the cytotoxicity of Pan-Ras-In-1. Although the effective concentrations to eliminate *S. aureus* were lower than the safety concentration determined by cytotoxicity assays, we believe that further structural optimization is warranted to enhance its bactericidal potency and reduce cellular toxicity. In combination with PELSA and molecular docking analyses, the TYR-146 site of the TarA protein appears to be the most likely binding site for Pan-Ras-In-1; however, further investigations are needed to verify this. Additionally, we noted that CidA was not detected by the PELSA assay. We believe this is because cell membrane-anchored target proteins, such as CidA, are not detectable by the PELSA method, a limitation also noted in the original developer’s publication.^29^ The cell lines used in this study were purchased from commercial vendors. Although all experiments were carried out with low-passage cells to mitigate phenotypic drift, the impacts of possible cell misidentification or unrecognized mycoplasma contamination cannot be ruled out.

## Resource availability

### Lead contact

Requests for further information and resources should be directed to and will be fulfilled by the lead contact, Fangyou Yu (wzjxyfy@163.com).

### Materials availability

This study did not generate new unique reagents.

### Data and code availability

• RNA-Seq data have been deposited at the NCBI Sequence Read Archive (SRA): "SRA: PRJNA1270757".
• The mass spectrometry proteomics data have been deposited at the ProteomeXchange Consortium via the iProX partner repository: "iProX: IPX0013716000".
• The raw data have been deposited in Mendeley Data: "Mendeley Data: https://www.doi.org/10.17632/8zdtv2w6rx.2".
• This paper does not report original code.
• The processed data and analysis scripts used in this study are available from the corresponding author upon request.
• Any additional information required to reanalyze the data reported in this paper is available from the lead contact upon request.

## Acknowledgments

This study was supported by the 10.13039/501100001809National Natural Science Foundation of China (grant no. 82302592).

## Author contributions

Conceptualization, F.Y., Y.S., and W.H.; methodology, W.H., Y.G., L.S., R.H., Y.H., X.Y., and C.Y.; investigation, W.H., B.W., and Y.S.; writing – original draft, W.H.; writing – review and editing, F.Y. and Y.S.; funding acquisition, Y.S.; resources, F.Y.; supervision, F.Y., Y.S., and B.W.

## Declaration of interests

The authors report no competing interests.

## STAR★Methods

### Key resources table

**Table:** 

REAGENT or RESOURCE	SOURCE	IDENTIFIER
**Bacterial and virus strains**		

SA113	Shang et al.^38^	An MSSA strain exhibiting an OXA MIC of 0.5 μg/mL
NCTC8325	Xiao et al.^39^	An MSSA strain exhibiting an OXA MIC of 0.5 μg/mL
Newman	Khalifa et al.^40^	An MSSA strain exhibiting an OXA MIC of 0.125 μg/mL
HG003	Sassi et al.^41^	An MSSA strain exhibiting an OXA MIC of 0.5 μg/mL
ATCC29213	Liu et al.^42^	An MSSA strain exhibiting an OXA MIC of <0.25 μg/mL
N315	Kuroda et al.^43^	An MRSA strain exhibiting an OXA MIC of 32 μg/mL
USA300 (LAC)	Moran et al.^44^	An MRSA strain exhibiting an OXA MIC of 32 μg/mL
MW2	Wang et al.^45^	An MRSA strain exhibiting an OXA MIC of 16 μg/mL
Mu50	Kuroda et al.^43^	An MRSA strain exhibiting an OXA MIC of 512 μg/mL
MSSA1	This study	A clinical MSSA isolate exhibiting an OXA MIC of 1 μg/mL
MSSA2	This study	A clinical MSSA isolate exhibiting an OXA MIC of 0.5 μg/mL
MSSA3	This study	A clinical MSSA isolate exhibiting an OXA MIC of 0.25 μg/mL
MSSA4	This study	A clinical MSSA isolate exhibiting an OXA MIC of 2 μg/mL
MSSA5	This study	A clinical MSSA isolate exhibiting an OXA MIC of 0.5 μg/mL
MSSA6	This study	A clinical MSSA isolate exhibiting an OXA MIC of 1 μg/mL
MSSA7	This study	A clinical MSSA isolate exhibiting an OXA MIC of 0.5 μg/mL
MSSA8	This study	A clinical MSSA isolate exhibiting an OXA MIC of 1 μg/mL
MSSA9	This study	A clinical MSSA isolate exhibiting an OXA MIC of 0.25 μg/mL
MSSA10	This study	A clinical MSSA isolate exhibiting an OXA MIC of 0.5 μg/mL
MSSA11	This study	A clinical MSSA isolate exhibiting an OXA MIC of 1 μg/mL
MSSA12	This study	A clinical MSSA isolate exhibiting an OXA MIC of 1 μg/mL
MSSA13	This study	A clinical MSSA isolate exhibiting an OXA MIC of 1 μg/mL
MSSA14	This study	A clinical MSSA isolate exhibiting an OXA MIC of 1 μg/mL
MSSA15	This study	A clinical MSSA isolate exhibiting an OXA MIC of 0.5 μg/mL
MSSA16	This study	A clinical MSSA isolate exhibiting an OXA MIC of 1 μg/mL
MRSA1	This study	A clinical MRSA isolate exhibiting an OXA MIC of 32 μg/mL
MRSA2	This study	A clinical MRSA isolate exhibiting an OXA MIC of 32 μg/mL
MRSA3	This study	A clinical MRSA isolate exhibiting an OXA MIC of >512 μg/mL
MRSA4	This study	A clinical MRSA isolate exhibiting an OXA MIC of >512 μg/mL
MRSA5	This study	A clinical MRSA isolate exhibiting an OXA MIC of >512 μg/mL
MRSA6	This study	A clinical MRSA isolate exhibiting an OXA MIC of 512 μg/mL
MRSA7	This study	A clinical MRSA isolate exhibiting an OXA MIC of 512 μg/mL
MRSA8	This study	A clinical MRSA isolate exhibiting an OXA MIC of 512 μg/mL
MRSA9	This study	A clinical MRSA isolate exhibiting an OXA MIC of 64 μg/mL
MRSA10	This study	A clinical MRSA isolate exhibiting an OXA MIC of 32 μg/mL
MRSA11	This study	A clinical MRSA isolate exhibiting an OXA MIC of 32 μg/mL
MRSA12	This study	A clinical MRSA isolate exhibiting an OXA MIC of 64 μg/mL
MRSA13	This study	A clinical MRSA isolate exhibiting an OXA MIC of 128 μg/mL
MRSA14	This study	A clinical MRSA isolate exhibiting an OXA MIC of 64 μg/mL
MRSA15	This study	A clinical MRSA isolate exhibiting an OXA MIC of 64 μg/mL
MRSA16	This study	A clinical MRSA isolate exhibiting an OXA MIC of 128 μg/mL
MRSA17	This study	A clinical MRSA isolate exhibiting an OXA MIC of >512 μg/mL
MRSA18	This study	A clinical MRSA isolate exhibiting an OXA MIC of 512 μg/mL
MRSA19	This study	A clinical MRSA isolate exhibiting an OXA MIC of 512 μg/mL
MRSA20	This study	A clinical MRSA isolate exhibiting an OXA MIC of 512 μg/mL
MRSA21	This study	A clinical MRSA isolate exhibiting an OXA MIC of 512 μg/mL
MRSA22	This study	A clinical MRSA isolate exhibiting an OXA MIC of 512 μg/mL
MRSA23	This study	A clinical MRSA isolate exhibiting an OXA MIC of 32 μg/mL
MRSA24	This study	A clinical MRSA isolate exhibiting an OXA MIC of 16 μg/mL
JP21	Shen et al.^46^	A clinical MSSA isolate exhibiting strong biofilm-producing capacity
MR273	Xiao et al.^39^	A clinical MRSA isolate exhibiting strong biofilm-producing capacity
MR383	This study	A clinical MRSA isolate exhibiting strong biofilm-producing capacity
SA113-▵*cidA*	This study	*cidA* deletion mutant
SA113-▵*cidA*-C	This study	*cidA* complemented mutant
NUTH-K2044	Wu et al.^47^	A strain of *K. pneumoniae* exhibiting high virulence
HS11286	Bi et al.^48^	A strain of *K. pneumoniae* harboring *bla*_KPC-2_
YFKP-97	Han, W. et al.^49^	A strain of *K. pneumoniae* harboring *bla*_KPC-12_
CRKP-1610	This study	A Carbapenem-Resistant *K. pneumoniae* clinical isolate
CRKP-1602	This study	A Carbapenem-Resistant *K. pneumoniae* clinical isolate
CRKP-1606	This study	A Carbapenem-Resistant *K. pneumoniae* clinical isolate
CRAB1	This study	A Carbapenem-Resistant *A. baumannii* clinical isolate
CRAB2	This study	A Carbapenem-Resistant *A. baumannii* clinical isolate
ABFK1	This study	An *A. baumannii* clinical isolate
ABFK2	This study	An *A. baumannii* clinical isolate
ABFK3	This study	An *A. baumannii* clinical isolate
ABFK4	This study	An *A. baumannii* clinical isolate
PAO1	Stover et al.^50^	A *P. aeruginosa* standard strain for research
PAFK1	This study	A *P. aeruginosa* clinical isolate
PAFK2	This study	A *P. aeruginosa* clinical isolate
PAKF3	This study	A *P. aeruginosa* clinical isolate
PAFK4	This study	A *P. aeruginosa* clinical isolate
PAFK5	This study	A *P. aeruginosa* clinical isolate
DH5α	Laboratory stock	*E. coli* isolates, clone host strain
DC10B	Laboratory stock	*E. coli* isolates, clone host strain

**Chemicals, peptides, and recombinant proteins**		

Tryptic soy broth (TSB) medium	Oxoid	CM0129
Mueller-Hinton II broth (cation-adjusted)	BD	212322
chloramphenicol	Solarbio	IC0320
anhydrotetracycline	Sigma-Aldrich	PHR3175
ampicillin	Sangon Biotech	A430258
vancomycin	MedChemExpress	HY-B0671
levofloxacin	MedChemExpress	HY-B0330
Dulbecco’s Modified Eagle’s Medium (DMEM)	Gibco	12491015
fetal bovine serum (FBS)	Procell	164210
Pan-Ras-In-1	TargetMol	T16432
SYTO9	Thermo Fisher	S34854
propidium iodide (PI)	Thermo Fisher	P3566
TB Green^TM^ Premix Ex Taq^TM^ II	Takara Bio	CN830S
PrimeSTAR® GXL DNA Polymerase	Takara Bio	R051A
solvent dimethyl sulfoxide (DMSO)	Sigma-Aldrich	D2653

**Critical commercial assays**		

Cell Counting Kit-8 (CCK8)	TargetMol	C0005
Rapid Bacterial Genomic DNA Isolation Kit	Sangon Biotech	B518225
Bacteria Total RNA Isolation Kit	Sangon Biotech	B518625
Evo M-MLV RT Kit with gDNA Clean	Accurate Biology	AG11705

**Deposited data**		

The RNA-Seq data	NCBI Sequence Read Archive (SRA)	PRJNA1270757
The mass spectrometry proteomics data	iProX partner repository	IPX0013716000
Raw data	Mendeley Data	https://doi.org/10.17632/8zdtv2w6rx.2

**Experimental models: cell Lines**		

Raw264.7	Sigma-Aldrich	CB_85062803

**Experimental models: organisms/strains**		

BALB/cA Mice	Shanghai Slac Laboratory Animal Co., Ltd.	BALB/cAnSlac

**Recombinant DNA**		

pKOR1	Bae et al.^51^	Gene replacement vector for *S. aureus* genes, Amp^r^, Cm^r^
pLi50	Han et al.^52^	*E. coli*- *S. aureus* shuttle vector, Amp^r^, Cm^r^
pKOR1-*cidA*	This study	pKOR1 derivative, for deletion of *cidA*
pLi50-*cidA*	This study	pLi50 derivative, for complementation of *cidA*

**Software and algorithms**		

GraphPad Prism (version 8.0.0)	GraphPad Software	https://www.graphpad.com/
R	R project	https://www.r-project.org

Amp^r^, ampicillin resistance; Cm^r^, chloramphenicol resistance; MRSA, methicillin-resistant *Staphylococcus. aureus*; MSSA, methicillin-sensitive *Staphylococcus. aureus*.

### Experimental model and study participant details

#### Bacterial strains, cell line, and culture conditions

The bacterial strains used in this study were either isolated from clinical samples or standard strains maintained in our laboratory. Detailed information on these strains was provided in key resources table. Unless otherwise noted, all strains were cultured in tryptic soy broth (TSB, Oxoid) medium at 37°C with shaking at 220 rpm. For antibiotic selection and plasmid maintenance, appropriate antibiotics were used at the following concentrations: for *S. aureus*, chloramphenicol (Solarbio) at 10 μg/mL, and anhydrotetracycline (Sigma-Aldrich) at 2 μg/mL; for *E. coli*, ampicillin (Sangon Biotech) at 100 μg/mL. The murine macrophage cell line Raw264.7 was brought from Sigma-Aldrich and cultured with Dulbecco’s Modified Eagle’s Medium (DMEM) supplemented with 10% (v/v) fetal bovine serum (FBS) at 37°C with 5% (v/v) CO_2_.

#### Animal

6-week-old female BALB/cAnSlac mice were purchased from Shanghai Slac Laboratory Animal Co., Ltd. (Shanghai, China). All the mice used in this study were maintained under pathogen-free conditions on a 12-h light–dark cycle, with free access to food and water. All animal procedures were approved by the Ethics Committee of the Shanghai Pulmonary Hospital of Tongji University School, Tongji University, Shanghai (Ethical no. K23-088Y).

### Method details

#### Antibacterial susceptibility testing against the ESKAPE pathogens

The antibacterial activity of Pan-Ras-In-1 (CAS number: 1835283-94-7; TargetMol, T16432) against ESKAPE pathogens was evaluated through the determination of MICs and MBCs in accordance with the standardized Clinical and Laboratory Standards Institute (CLSI) guidelines (CLSI2021-M100-ED31). Briefly, the exponential-phase bacterial suspension was diluted to 1.5 × 10^6^ CFU/mL in Mueller-Hinton II broth (cation-adjusted, BD). Subsequently, Pan-Ras-In-1 was serially diluted 2-fold in 96-well plates, and 100 μL bacterial dilution was distributed. After incubation at 37°C for 24 h, MIC values were determined as the lowest compound concentration that inhibited visible bacterial growth. Furthermore, 100 μL of liquid was removed from wells without visible bacterial growth and spread onto Mueller-Hinton agar (MHA) plates for a further 24 h of culture to determine the MBCs.

#### Time-kill kinetics study

The bacterial solutions in the exponential phase were diluted in sterile phosphate-buffered saline (PBS, Sangon, Shanghai) and treated with Pan-Ras-In-1, vancomycin, or levofloxacin at serial concentrations. The bacterial solutions were then diluted and spread onto plates to determine CFU at different time points.

#### Live/dead bacterial staining assay

Overnight cultures of *S. aureus* were diluted 1:100 in fresh TSB medium and incubated in glass-bottom cell culture dishes (Biosharp). The supernatants were discarded after incubating at 37°C for 12 h, and the dishes were washed with sterile PBS three times. Subsequently, the solutions containing 4-fold MIC of Pan-Ras-In-1, vancomycin, or levofloxacin were added to the dishes to treat the adhesive bacteria. After 4 h of treatment, the solutions were discarded, and 500 μL PBS containing 0.02% SYTO9 (Thermo Fisher) and 0.067% propidium iodide (PI, Thermo Fisher) was used to stain alive and dead bacteria, respectively, in the dark. Then, the samples were scanned using the confocal laser scanning microscopy (TCS SP5; Leica) with a 63 ×1.4 numerical aperture oil immersion lens objective.

#### Intracellular bactericidal activity assay

The ability of Pan-Ras-In-1 to kill the bacteria inside host eukaryotic cells was determined as described in the previous paper.^53^ In this assay, we used the murine macrophage cell line Raw 264.7 to establish intracellular infection with *S. aureus*. Subsequently, intracellular *S. aureus* was exposed to Pan-Ras-In-1 at a concentration of 2-fold MIC, a dosage determined to be non-cytotoxic to host cells. To evaluate the intracellular bactericidal efficacy of Pan-Ras-In-1, vancomycin and levofloxacin were used as comparators, and the untreated group served as the negative control. Following a 2-h incubation, Raw 264.7 cells were enzymatically dissociated with 200 μL of trypsin-EDTA solution for 3 min and lysed with 0.05% Triton X-100 for 5 min. Finally, the bacterial CFU was determined by plating serial dilutions of cell lysates on TSA plates, and the killing rate was calculated using the following formula: Survival rate (%) = CFU_sample_/CFU_control_ × 100. Experiments were repeated three times.

#### Biofilm prevention assay

Overnight cultures of *S. aureus* were diluted 1:100 in fresh TSBG medium (TSB medium supplemented with 0.5% glucose) containing serially diluted concentrations of Pan-Ras-In-1. The bacterial suspensions were then aliquoted into 96-well polystyrene microtiter plates and incubated at 37°C for 24 h. Following separation of the supernatant and attached bacteria, the wells were gently washed three times with PBS. Subsequently, methanol was used to fix the bacterial cells for 10 min, and 1% crystal violet was used to stain the biofilms for an additional 10 min. Finally, the biofilms were dissolved in 30% acetic acid, and OD_600_ was measured using a microplate reader (Hiwell-Diatek Instruments).

#### Induced resistant mutant generation studies

In this assay, *S. aureus* SA113 and USA300 (LAC) were subjected to serial passaging in the presence of 1/2 MIC concentrations of Pan-Ras-In-1 and levofloxacin to induce drug resistance. MIC values were monitored at each passage, and results were presented as fold increases relative to the initial MIC.

#### Cell viability assay

The CCK-8 assay was used to evaluate cell viability. In brief, the Raw 267.4 cells (5 × 10^4^ cells/well) were treated with serial concentrations of Pan-Ras-In-1 (2.9–8.5 μg/mL) at 37°C with 5% (v/v) CO_2_ for 24 h, and the group treated with solvent dimethyl sulfoxide (DMSO, Sigma-Aldrich) served as a negative control. Subsequently, the Cell Counting Kit-8 (CCK8) (TargetMol, C0005) was used to evaluate cell viability according to the manufacturer’s instructions, and OD_450_ values were recorded using a microplate reader (Hiwell-Diatek Instruments). Finally, the cell viability (%) was calculated using the following formula: cell viability (%) = (OD_450_ of the compound-treated well - OD_450_ of the blank well)/(OD_450_ of the non-compound-treated well - OD_450_ of the blank well) × 100. The IC_50_ was calculated, representing the compound concentration that induces a 50% reduction in cell viability. Additionally, the SI was calculated as IC50/MIC.

#### Hemolytic activity assay

In this assay, 100 μL PBS containing serial concentrations of Pan-Ras-In-1 (2.25–176 μg/mL) was mixed with 900 μL of 2% (v/v) fresh healthy adult blood cells (obtained from volunteers who provided written informed consents for blood usage in research investigations). After incubating at 37°C for 1 h, the supernatants were collected by centrifugation at 4,000 rpm for 5 min, and the absorbance of the supernatant at 600 nm was recorded. PBS and Triton X-100 served as negative and positive controls, respectively. Hemolysis ratio was calculated as: Hemolysis (%) = (OD_600_, treated - OD_600_, negative)/(OD_600_, positive - OD_600_, negative) × 100. The HC_50_ was defined as the compound concentration inducing 50% red cell lysis, and the SI was calculated as HC50/MIC.

#### RNA-seq and data analysis

This assay was performed with minor modification as previously described.^52^ *S. aureus* SA113 overnight cultures were diluted 1:200 in TSB with or without 1.8 μg/mL (0.8 × MIC) Pan-Ras-In-1, then cultured for 6 h to the logarithmic growth phase. Total RNA was extracted using the Bacterial Total RNA Isolation Kit (Sangon Biotech), and then mRNA was purified for cDNA library construction. Library quality was checked with the Agilent Bioanalyzer 2100 system. After cluster generation, RNA sequencing was carried out on an Illumina NovaSeq-PE150 platform. Clean reads were mapped to the *S. aureus* NCTC8325 genome (GenBank: CP000253) using Bowtie 2 (version 2.2.3),^54^ allowing two mismatches. Read counts were normalized by the edgeR package, and DEGs were identified using DESeq2 R package (version 1.18.0) with threshold of FDR ≤0.05 and |log_2_(fold change)| ≥ 1. KEGG and GO enrichment analyses were performed using KOBAS and Goseq, respectively.

#### RNA isolation, cDNA synthesis, and RT-qPCR analysis

The experimental procedure was slightly modified from a previously published method.^52^ *S. aureus* cultures were diluted 1:200 in TSB medium with or without 1.8 μg/mL Pan-Ras-In-1 for 6 h. After centrifugation, bacterial pellets were resuspended in TE buffer supplemented with lysozyme and lysostaphin, followed by incubation at 37°C for cell lysis. Total RNA was isolated using a Bacteria Total RNA Isolation Kit (Sangon Biotech) following the manufacturer’s protocol. Subsequently, RNA was reverse-transcribed into cDNA with a cDNA synthesis kit (Accurate Biology), and RT-qPCR was conducted using TB Green™ Premix Ex Taq™ II (Takara) on a QuantStudio™ 5 System. Corresponding primer sequences are provided in Table S4. Relative gene expression levels were calculated using the 2^−ΔΔCT^ method with *gyrB* as the internal reference gene. All reactions were performed in triplicate biological and technical replicates.

#### Triton X-100-induced autolysis assay

The assay was carried out with minor adjustments based on a previous report.^52^ *S. aureus* cultures were diluted 1: 100 in TSB containing 1M NaCl (with or without Pan-Ras-In-1), and cultured at 37°C with shaking (220 rpm) until the cultures reached an OD_600_ of 0.6–0.8. After being washed with ice-cold sterile water for three times, the bacteria were resuspended in 0.05 M Tris-HCl (pH 7.2) supplemented with 0.05% (v/v) Triton X-100 to an OD_600_ of 1.0. After incubation at 37°C with shaking (220 rpm), the OD_600_ value was recorded every 30 min. All data were obtained from three independent experiments and are presented as the mean ± standard deviation.

#### Transmission electron microscopy (TEM)

*S. aureus* was cultured to the mid-log phase and treated with 1.8 μg/mL Pan-Ras-In-1 or DMSO. Bacterial cells were harvested gently and fixed overnight at 4°C with 2.5% glutaraldehyde in phosphate buffer, then post-fixed with 1% OsO_4_ and stained with uranyl acetate. After dehydration in a graded ethanol series and embedding in resin, ultrathin sections were prepared and stained with lead citrate. Samples were finally observed and imaged using a Tecnai F20 G2 transmission electron microscope at 100 kV.

#### Intracellular compounds leakage measurement

The bacterial DNA leakage experiment was performed as previously described, with slight modifications.^55^ Overnight cultures of *S. aureus* were diluted 1:200 in fresh TSB medium and incubated at 37°C with shaking (220 rpm) for 6 h to reach the logarithmic growth phase. The bacterial cultures were adjusted to a density of 1.5 × 10^8^ CFU/mL and subsequently resuspended in PBS. Pan-Ras-In-1 was added to the bacterial suspensions to achieve a final concentration of 4-fold MIC. Afterward, the mixture was incubated at 37°C with shaking (220 rpm) for 4 h, followed by centrifugation at 4,000 rpm for 5 min. After filtering the supernatant through a 0.22-μm-pore-size polyether sulfone membrane to remove bacterial cells, the DNA concentration was measured by absorbance at 260 nm using a NanoDrop 2000 spectrophotometer (Thermo Fisher).

#### Construction of *S. aureus cidA* gene deletion and complemented mutants

The *cidA* deletion mutant was generated using the allelic exchange method. Briefly, the upstream (826 bp) and downstream (1147 bp) fragments flanking *cidA* were amplified from *S. aureus* SA113 genomic DNA and fused by overlap extension PCR (PrimeSTAR® GXL DNA Polymerase, Takara). The fused fragment was cloned into the pKOR1 vector (10, 374 bp),^51^ and the resulting recombinant plasmid was verified by sequencing after being transformed into *E. coli* DH5a and DC10B. The construct was then electroporated into *S. aureus* SA113 to achieve allelic replacement. Finally, the *cidA* mutant strain was confirmed by PCR, DNA sequencing, and RT-qPCR (Figure S4). For constructing complemented mutant, the full-length *cidA* gene with its native promoter (1, 000 bp) was amplified and cloned into the pLi50 vector (5, 505 bp). After sequence verification, the recombinant plasmid was introduced into the ▵*cidA* mutant via electroporation. Successful complementation was verified by RT-qPCR (Figure S4).

#### Peptide-centric local stability assay (PELSA)

The peptide-centric local stability assay was carried out following the protocol of Li et al.^29^ *S. aureus* SA113 cells were ground in liquid nitrogen and lysed in ice-cold lysis buffer containing protease inhibitors. After resuspension, the total proteome was extracted and adjusted to 1 mg/mL. Aliquots of the lysate were incubated with 22.5 μg/mL Pan-Ras-In-1 solution or DMSO solvent control at 25°C for 30 min in parallel for four replicates. Native tryptic digestion was performed briefly and then terminated with guanidine hydrochloride. After reduction and alkylation, the digested peptides were filtered, washed, and desalted using C18 stage tips. The samples were then vacuum-dried, dissolved in 0.1% formic acid, and subjected to liquid chromatography-tandem mass spectrometry (LC-MS/MS).

#### LC-MS/MS analysis

The peptide samples derived from PELSA assays were analyzed using an Orbitrap Eclipse mass spectrometer (Thermo Fisher) coupled with an Easy-nLC1200 nano-flow system (Thermo Fisher). Approximately 0.5 μg peptides were separated on a C18 column (Easypept) at 55°C, with a 60-min linear gradient using standard aqueous/organic mobile phases containing 0.1% formic acid. Data were acquired in data-independent acquisition mode with optimized electrospray parameters. MS1 spectra were recorded over a mass range of 400–1000 m/z, while DIA scans were collected using variable isolation windows with HCD fragmentation energy set to 28%. All spectra were acquired at high resolution with optimized ion injection times.

#### Proteomic data analysis

The MS/MS data were analyzed for protein identification and quantification using Spectronaut (29) (Biognosys AG, v.18.0) and DIA-NN (30) (v.2.1.0, Academia). A nonredundant UniProt *S. aureus* (strain NCTC 8325/PS 47) database containing 2,889 proteins (downloaded in 2025) was imported as a FASTA file for directDIA searching with default parameters. PELSA-Decipher^56^ was used for the processing and interpretation of PELSA data.

#### Molecular docking

Molecular docking was performed using AutoDock 4.2.6. The PDB file of target protein TarA (UniProt ID: Q2G2L3) was downloaded from AlphaFold Protein Structure Database, while the three-dimensional structure of Pan-Ras-In-1 (PubChem CID: 102004330) was obtained from Pubchem Compound Database. The protein structure was prepared by removing water molecules and adding polar hydrogens using AutoDock tools. Additionally, the docking parameters were optimized using a genetic algorithm, and the model with the lowest binding energy within the maximum cluster of docked conformations was selected as the representative structure. Finally, the structure was visualized by PyMOL.

#### Extraction and analysis of the polymerization degree of *S. aureus* wall teichoic acid

The wall teichoic acid was extracted and analyzed as previously described in a study with slight modifications.^57^ Briefly, 200 mL cultures of *S. aureus* strains at the early exponential phase (OD_600_ = 0.5) were harvested with centrifugation at 5,000 × g for 10 min. The pellet was resuspended in 50 mM MES buffer (pH 6.5) to normalize the similar OD_600_ value, then centrifuged at 5,000 × g for 10 min to collect cells. After resuspension in 20 mL MES buffer (pH 6.5) containing 4% (w/v) SDS, the pellet was incubated in boiling water for 1 h. Subsequently, the pellets were resuspended and collected in 2 mL of 50 mM MES buffer (pH 6.5) containing 2% (w/v) NaCl, and then in 2 mL of 50 mM MES buffer (pH 6.5). Afterward, the pellets were suspended in 2 mL Tris-HCl buffer (pH 8.0) containing 0.5% (w/v) SDS, and 20 μL proteinase K solution (20 mg/mL) was added to degrade cell wall-associated proteins. This process was incubated at 50°C with shaking at 1,000 rpm for 4 h. Subsequently, the pellets were collected by centrifugation at 12,000 × g for 5 min and washed with 2 mL of 50 mM MES buffer (pH 6.5) containing 2% (w/v) NaCl three times. Finally, the pellets were resuspended in 1 mL NaOH solution (0.1 mM) and incubated at 25°C with shaking at 1,000 rpm for 16 h. The supernatant containing extractable WTA was collected with centrifugation at 12,000 × g for 5 min and used for further analysis. The polymerization degree of WTA at different *S. aureus* cell surfaces was analyzed through their different migration in a 15% native polyacrylamide gel electrophoresis (PAGE), and further detection was performed by using Alcian blue -silver staining. This experiment was repeated three times.

#### Murine bacteremia model

6-week-old female BALB/cAnSlac mice were randomly divided into three groups. The overnight culture of *S. aureus* USA300(LAC) was diluted 1:200 in fresh TSB medium and incubated at 37°C with shaking (220 rpm) for 6 h to reach the logarithmic growth phase. After being harvested by centrifugation at 4,000 × *g* for 5 min, the bacteria were washed twice and adjusted to 1 × 10^9^ CFU/mL. Then, 1 × 10^8^ CFU was injected into mice via the tail vein. At 1-h post-infection, 5 mg/kg of Pan-Ras-In-1 or vancomycin was administered to the treatment group, with DMSO as the control. At 9 h post-infection, the mice were treated for a second time with 5 mg/kg Pan-Ras-In-1 or vancomycin. Mice were euthanized at 24 h, and visceral organs, including heart, liver, spleen, lung, and kidney, were aseptically excised and homogenized in sterile PBS. Bacterial loads in the specific organs were determined by serial dilution and plating on TSB plates. Additionally, hematoxylin and eosin (H&E) staining was performed on each organ.

#### Murine skin abscess model

The murine skin abscess model was utilized to further evaluate the bactericidal capability of Pan-Ras-In-1 *in vivo*. 6-week-old female BALB/cAnSlac mice were removed of their back hair and assigned randomly into three groups, each containing seven mice: a group with *S. aureus* infection only, a positive control group treated with vancomycin (0.5 mg/kg), and a group treated with a similar dosage of Pan-Ras-In-1 (0.5 mg/kg). The mice were housed in a specific pathogen-free facility, and a libitum diet was provided. Overnight cultures of *S. aureus* were diluted 1:200 in fresh TSB medium and incubated at 37°C with shaking (220 rpm) for 6 h to the logarithmic growth phase. After centrifugation at 4,000 × g for 5 min, the bacteria were washed twice and adjusted to a turbidity equivalent to a 0.5 McFarland standard in PBS. Then, 100 μL bacterial suspension (1.5 × 10^8^ CFU/mL) was injected subcutaneously into the shaved flank to induce subcutaneous abscesses. At 2 h post-infection, 100 μL vancomycin, Pan-Ras-In-1 or DMSO vehicle was administered by subcutaneous injection at the same site where bacteria were injected. Abscess sizes were measured for three days using calipers, according to the formula A = *Π* × (*L* × *W*). On the third day, the infected skin was carefully dissected under aseptic conditions, and the bacterial load in the abscess was determined by serial dilutions and spotted on TSA plates. Furthermore, skin tissues were harvested and subjected to H&E staining.

### Quantification and statistical analysis

GraphPad Prism version 8.00 software was used to conduct statistical analysis. A two-tailed Student’s *t* test was used to analyze the statistical difference between two groups, and one-way analysis of variance (ANOVA) was used to analyze the statistical difference between multiple groups after passing the normal distribution test. Error bars in the figures represent the standard deviation of the dataset (mean ± standard deviation). ∗*p* < 0.05, ∗∗*p* < 0.01, ∗∗∗*p* < 0.001, ∗∗∗∗*p* < 0.0001.

## Supplemental information

Document S1. Figures S1–S4

Table S1. The MIC and the MBC of Pan-Ras-In-1 for pathogens commonly detected in the clinic

Table S2. Summary of genes upregulated in the Pan-Ras-In-1 treatment group

Table S3. Summary of genes downregulated in the Pan-Ras-In-1 treatment group

Table S4. Primers used in this study
