# Supplementary material for: Pan-Ras-In-1 triggers cell wall lysis to eradicate Staphylococcus aureus
Source: iScience. 2026 Aug 28;29(9):117291. doi: 10.1016/j.isci.2026.117291 (PMC13545390; doi:10.1016/j.isci.2026.117291)
Supplement: Supplementary file 1 [file mmc1.pdf]

iScience, Volume *29*

Supplemental information

Pan-Ras-In-1 triggers cell wall lysis to eradicate *Staphylococcus aureus*

Weihua Han, Yinjuan Guo, Li Shen, Yu Huang, Xinru Yuan, Bingjie Wang, Can Yang, Rongrong Hu, Yongpeng Shang, and Fangyou Yu

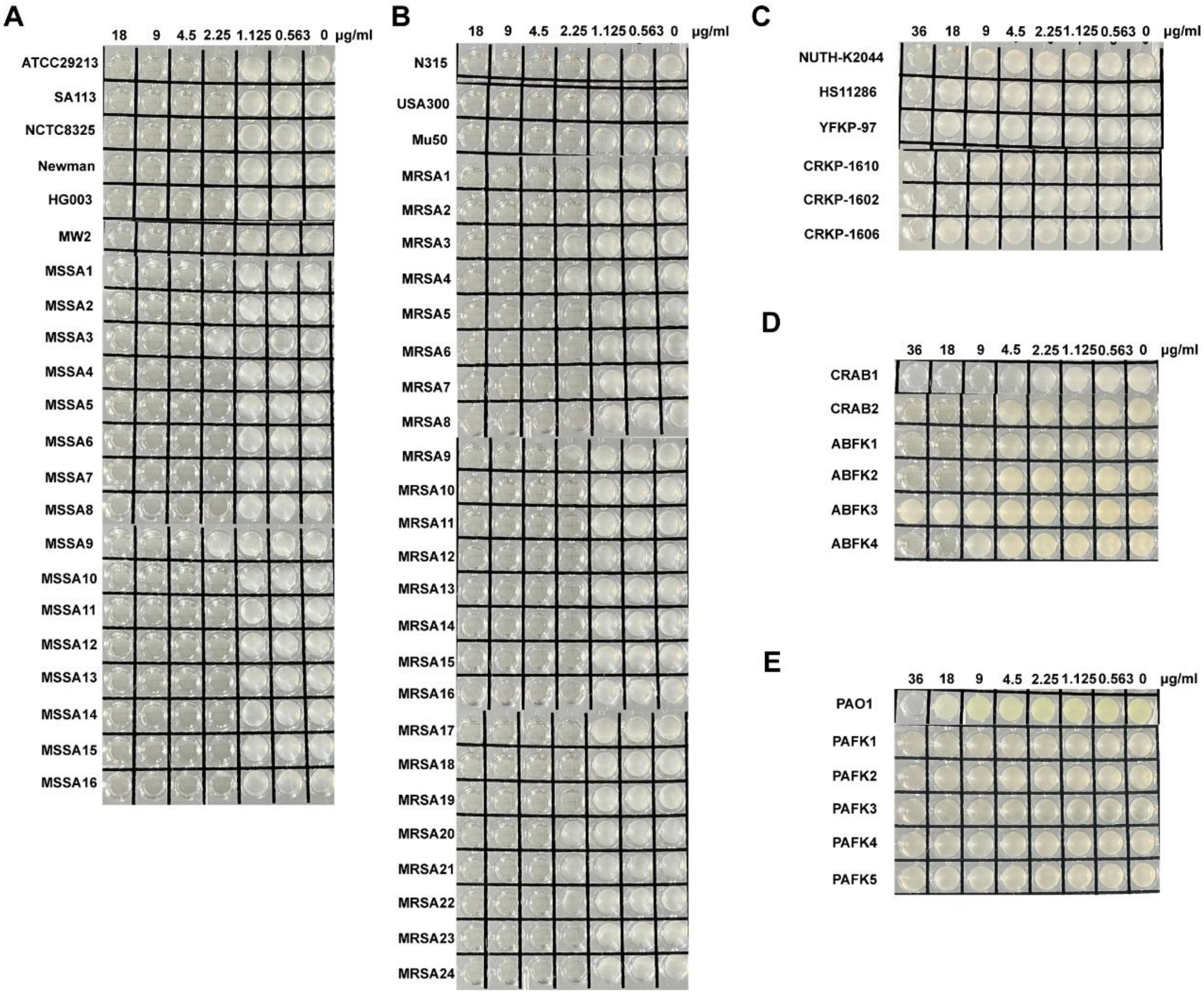

Figure S1 The MIC results of Pan-Ras-In-1 against the methicillin-sensitive *S. aureus* (MSSA) (A), methicillin-resistant *S. aureus* (MRSA) (B), *Klebsiella pneumonia* (C), *Acinetobacter baumannii* (D), and *Pseudomonas aeruginosa* (E).

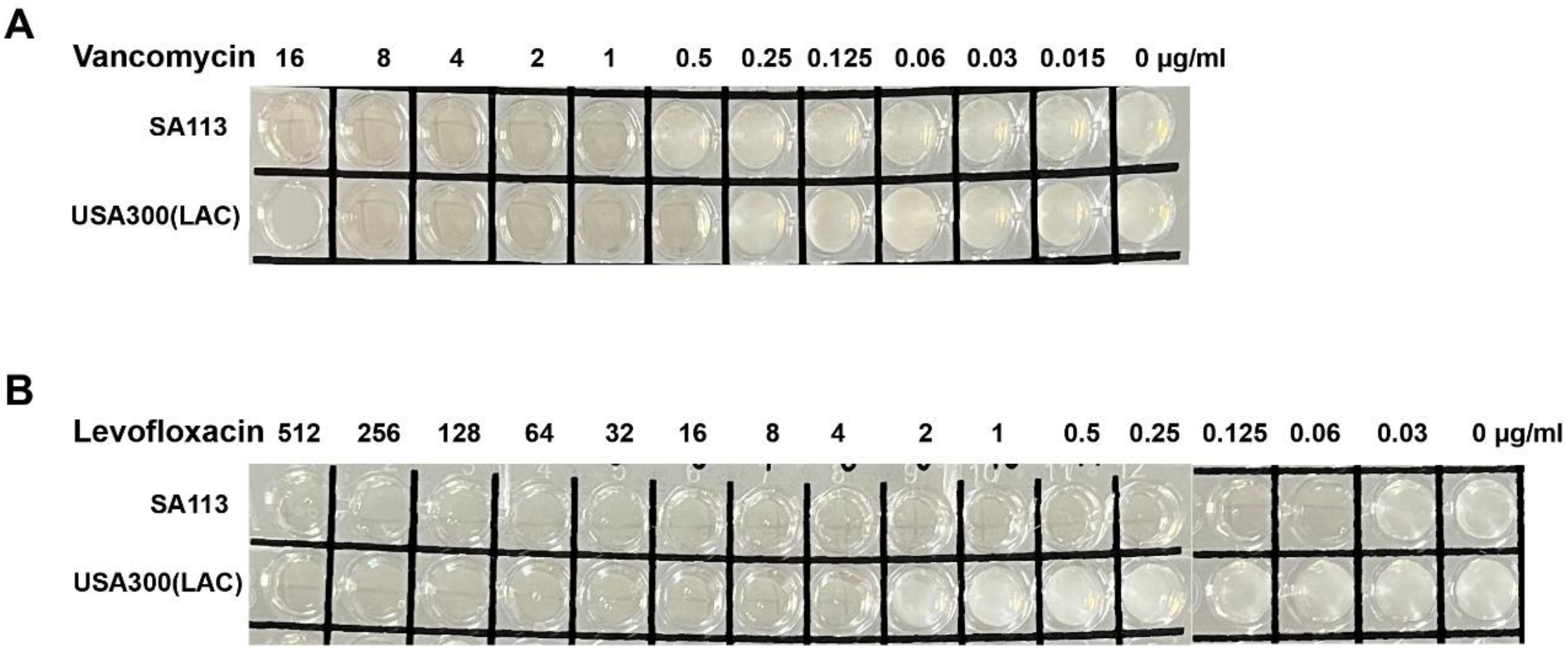

Figure S2 The MIC results of vancomycin (A) and levofloxacin (B) against the *S. aureus* SA113 and USA300(LAC).

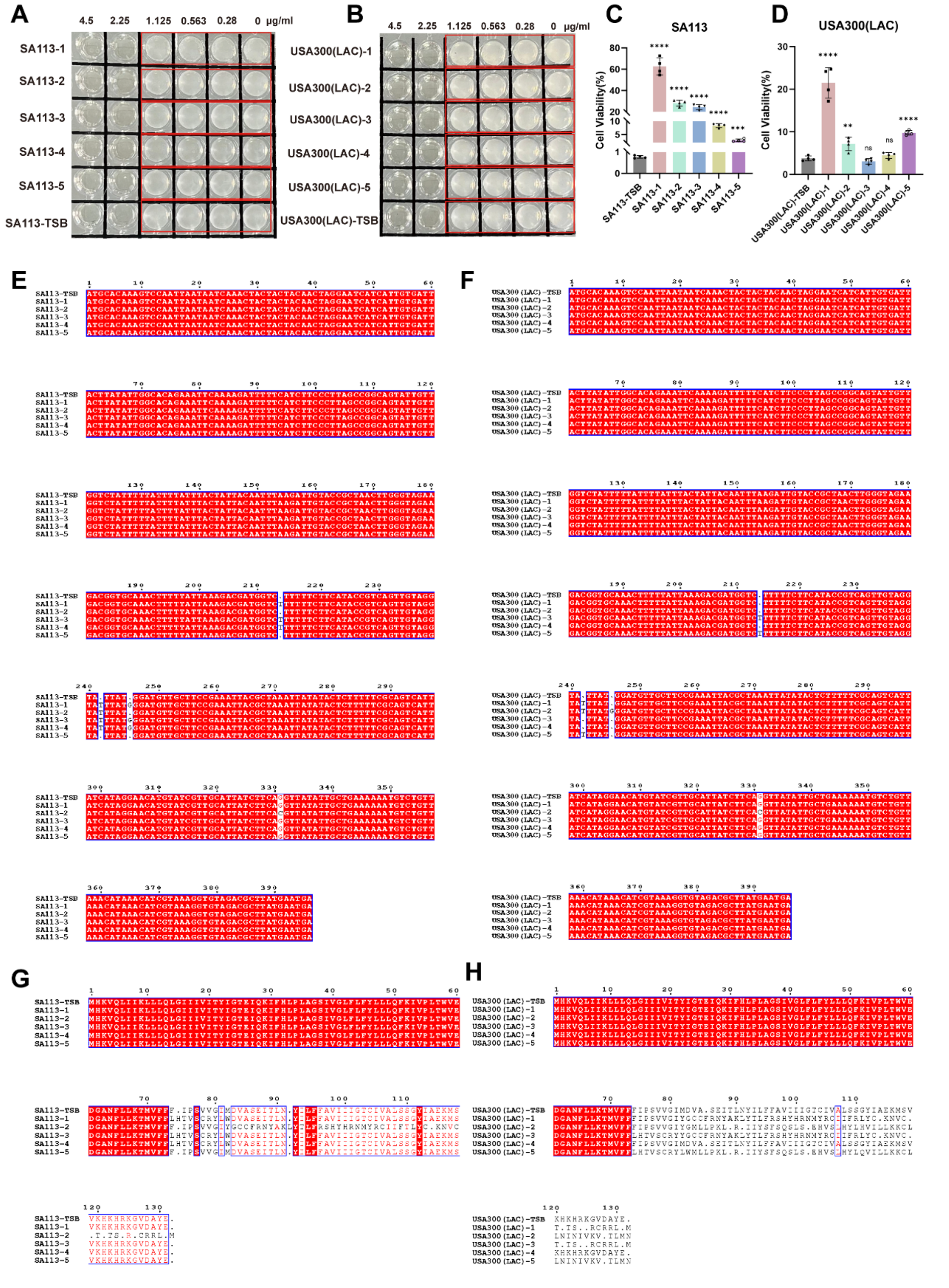

Figure S3 The mutation sites of *cidA* in Pan-Ras-In-1 tolerant strains yielded by in vitro resistance induction. (A) and (B) The MICs results of Pan-Ras-In-1 against induction strains. (C) and (D) The ability of strains to resist the bactericidal activity of Pan-Ras-In-1. (E) and (F) The nucleotide sequence alignments of *cidA* in relevant strains. (G) and (H) The amino acid sequence alignments of CidA in relevant strains. A two-tailed Student's t-test was used to analyze the statistical difference between two groups after passing the normal distribution test (C and D). Error bars in the figures represent the

standard deviation of the dataset (mean ± standard deviation). ns, not significant, *P < 0.05, **P < 0.01, ***P < 0.001, ****P < 0.0001.

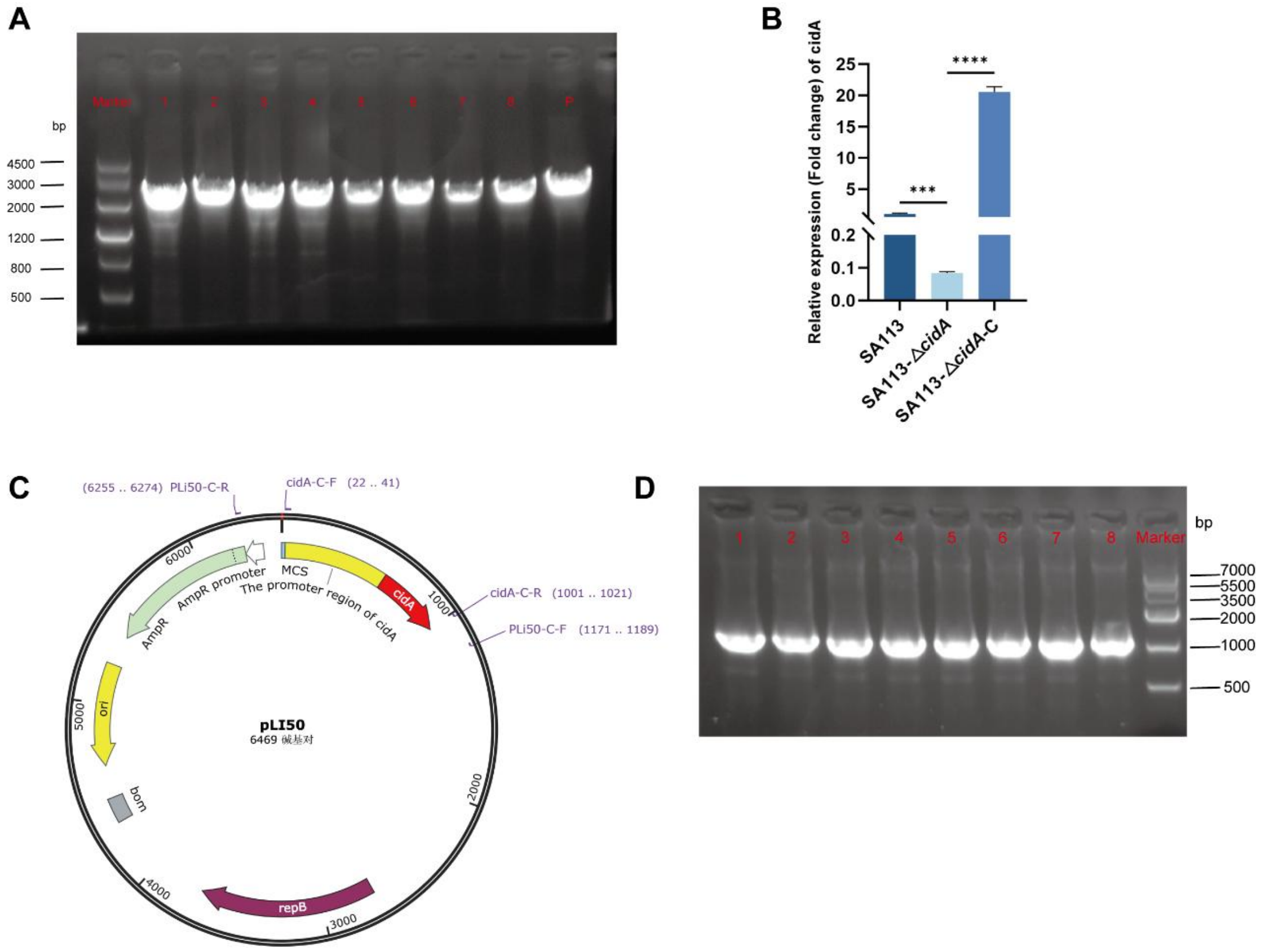

Figure S4 Construction of *S. aureus cidA* gene deletion and complementary mutants. (A) Confirm the deletion of the *cidA* gene by PCR with primers *cidA*-out-F and *cidA*-out-R. Genomic DNA from the wild-type strain was used as a positive control (the ninth lane labeled "P"). The lower band in the first lane indicated successful construction of the *cidA* knockout strain. (B) Confirm the successful construction of the *cidA* deletion and complementary strains by RT-qPCR. (C) Vector map for the *cidA* complementary plasmid. (D) Confirm the construction of *cidA* complementary strain by PCR with primers pLi50-C-F and pLi50-C-R.
